# Nutraceutical Activity in Osteoarthritis Biology: A Focus on the Nutrigenomic Role

**DOI:** 10.3390/cells9051232

**Published:** 2020-05-16

**Authors:** Stefania D’Adamo, Silvia Cetrullo, Veronica Panichi, Erminia Mariani, Flavio Flamigni, Rosa Maria Borzì

**Affiliations:** 1 Department of Medical and Surgical Sciences, Alma Mater Studiorum-University of Bologna, 40126 Bologna, Italy; stefania.dadamo2@unibo.it (S.D.); erminia.mariani@unibo.it (E.M.); 2 Laboratory of Immunorheumatology and Tissue Regeneration, IRCCS Istituto Ortopedico Rizzoli, 40136 Bologna, Italy; rosamaria.borzi@ior.it; 3 Department of Biomedical and Neuromotor Sciences, Alma Mater Studiorum-University of Bologna, 40126 Bologna, Italy; veronica.panichi2@unibo.it (V.P.); flavio.flamigni@unibo.it (F.F.)

**Keywords:** nutraceuticals, osteoarthritis, nutrigenomics, inflammation, senescence, extracellular matrix remodeling

## Abstract

Osteoarthritis (OA) is a disease associated to age or conditions that precipitate aging of articular cartilage, a post-mitotic tissue that remains functional until the failure of major homeostatic mechanisms. OA severely impacts the national health system costs and patients’ quality of life because of pain and disability. It is a whole-joint disease sustained by inflammatory and oxidative signaling pathways and marked epigenetic changes responsible for catabolism of the cartilage extracellular matrix. OA usually progresses until its severity requires joint arthroplasty. To delay this progression and to improve symptoms, a wide range of naturally derived compounds have been proposed and are summarized in this review. Preclinical in vitro and in vivo studies have provided proof of principle that many of these nutraceuticals are able to exert pleiotropic and synergistic effects and effectively counteract OA pathogenesis by exerting both anti-inflammatory and antioxidant activities and by tuning major OA-related signaling pathways. The latter are the basis for the nutrigenomic role played by some of these compounds, given the marked changes in the transcriptome, miRNome, and methylome. Ongoing and future clinical trials will hopefully confirm the disease-modifying ability of these bioactive molecules in OA patients.

## 1. Introduction

Osteoarthritis (OA) is a degenerative disorder that affects diarthrodial joints (hand, knee, hip) mainly in the elderly, involving all districts: bone, synovium, meniscus, ligaments/tendons, and articular cartilage. Symptoms include pain, stiffness mainly in the morning, difficulty in joint movement, and physical disability leading to impediments in the execution of daily domestic and work activities. Indeed, the burden of OA includes not only impact on the individual, but also on the healthcare system and society, heavily influencing the national economy [1]. To date, pharmacotherapy for OA is mainly limited to analgesic and/or nonsteroidal anti-inflammatory drugs (NSAIDs) that counteract symptoms, but long-term use of which leads to gastrointestinal, renal, and cardiovascular side effects [2]. Moreover, these drugs lead at best to pain and inflammation reduction. Despite some promising but inconclusive results reported for cyclooxygenase 2 (COX-2) inhibitors and reviewed in Nakata et al. [3], NSAIDs are unable to slow progression or counteract the pathophysiology of the disease. Recent findings suggest that a useful alternative approach may be represented by food-derived compounds that, when supplemented in a patient’s diet, can prevent disease outcome, modify pathophysiological events, and even contribute to improved patient response to pharmacotherapy. Furthermore, their employment in clinical practice can be highly recommended for long periods [4] since they generally show a lack of confirmed side effects, with some exceptions [5], as some supplements are suspected to have adverse effects associated with long-term use (such as omega 3 fatty acids, which may influence wound-healing, hemostasis, and altered immune function due to effects on leukotriene B4).

Therefore, the interest of the scientific community is focused on basic and applied research to unveil the potential of diet-derived molecules to improve the quality of life or reverse the pathology of OA sufferers. “Nutraceutical” is a term that combines the words “nutrition” and “pharmaceutical”, and refers to a food or a food-derived bioactive component for which beneficial effectiveness has been scientifically demonstrated [6]. More than 17% of OA-affected patients use non-prescribed dietary supplements, thus showing an increased interest in complementary and alternative medicine for the management of the disease [7]. This is in keeping with developments in other areas of medicine; natural products are gaining an increasingly critical role in the discovery and development of numerous drugs for the treatment of various diseases such as cancers [8,9,10] as their mechanisms of action are coming to light. This review focuses on the role of nutraceuticals in the biology of OA, and in particular on their ability to influence gene expression of known OA markers and other factors implicated in chondrocyte homeostasis. The literature survey was performed by scanning the search engine PubMed by using the key words “osteoarthritis” or “chondrocytes” or “cartilage” and “nutraceutical” or “natural compounds” and/or the name of the specific compound of interest. The papers selected for the review were those that showed evidence of the nutraceutical effect on gene expression (i.e., nutrigenomic effects). Clinical trials were excluded as they were previously discussed in other reviews and beyond the scope of our paper, with the exception of a few studies pointing at the bioavailability of a nutraceutical or its usefulness as an "add-on" to conventional therapy.

## 2. Osteoarthritis Pathophysiology

Molecular evidence collected in recent decades has shed light on the mechanisms responsible for OA onset, thus changing its perception from a simple degenerative “wear and tear” disease to a progressively degenerative joint derangement sustained by inflammatory networks [11]. At the same time, its belonging to the group of age-associated diseases has increased the awareness that the homeostasis of most post-mitotic tissues is pivotal in preventing their functional failure. The knowledge-based use of interventions targeted at molecularly tuning selected signaling pathways with a role in OA pathogenesis might represent a safe route to rescue the functionality of the joint tissue. 

The onset of OA is facilitated by age or by conditions that precipitate cartilage aging (such as obesity and metabolic syndrome [12]) of a tissue that, however, will inevitably undergo marked changes of its molecular organization [13]. Accumulation of advanced glycation end-products (AGEs) in cartilage is a non-enzymatic modification of collagen proteins that is strictly correlated with aging [14]. AGE accumulation in collagen is responsible for both the loss of the tissue’s mechanical properties and for activating the chondrocyte receptors for AGEs (RAGEs), thus inducing nuclear factor kappa-light-chain-enhancer of activated B cells (NF-κB) activation. Aging and OA are associated with a status of systemic, sterile, and low-grade inflammation called “inflammaging” [15]. This condition is triggered by receptor (RAGEs and Toll like receptors) recognition of the so-called danger-associated molecular patterns (DAMPs) that at the joint level are mostly represented by cartilage matrix fragments or endogenous molecules (HMGB1, S100) released after damage of tissues or cells [16].

Most of our knowledge of the pathophysiology of OA derives from matching findings in cartilage obtained from tissue arthroplasty and findings obtained from studies of surgically induced OA performed in animal models [17]. Among the latter, the destabilization of the medial meniscus (DMM) performed in mice has become the method of choice for validation of OA target genes with functional genomics [18,19]. This approach has indicated a critical role of interleukin (IL)-1β and of A Disintegrin and Metalloproteinase with Thrombospondin motifs (ADAMTS)-5 in OA pathogenesis, thus showing that inflammation and extracellular matrix (ECM) remodeling have a pivotal role in this joint disease. 

Healthy articular cartilage is a post-mitotic tissue actively kept in a defined “differentiation window” by molecular constraints that are lost upon OA onset [13]. Therefore, when loss of maturational arrest occurs, OA chondrocytes progress into their default route, i.e., hypertrophy and terminal differentiation, as confirmed by an increased expression of Runt-related transcription factor (RUNX)-2 and vascular endothelial growth factor (VEGF), markers of chondrocyte hypertrophy and angiogenesis, respectively [20]. As in the process of chondrogenesis [21], this event is accompanied by a profound remodeling of major components of the ECM: aggrecan and collagen 2, with elicitation of bioactive cleavage peptides able to further boost the degradation process [22,23]. Articular cartilage ECM has a very ordered extracellular matrix that can be distinguished into territorial (perichondral) and interterritorial matrix. The first cleavage is at the level of aggrecan (aggregates of proteoglycans and hyaluronic acid), exerted by the so-called aggrecanases (aggrecanase-1 or ADAMTS-4 and aggrecanase-2 or ADAMTS-5). ADAMTS-4 is induced in chondrocytes by acute inflammatory stimuli [24], but ADAMTS-5 has a higher activity [25] and, in the early phase of the disease, primes the initial ECM-degrading event that allows the access of collagenases to collagen 2. Its pivotal role is confirmed by the effectiveness of counteracting OA progression in DMM models by targeting this catabolic enzyme using small interfering RNAs [26] or antibodies [27].

Cleavage of collagen 2 is the “point of no return” in cartilage degradation. It is mediated by collagenases, a class of proteolytic enzymes able to cleave collagen at ¼ and ¾ of its length, thus exposing the epitope C1,2C (= collagen 1 and 2 cleavage), i.e., a α-chain fragment that further enhances cartilage degradation. This group of enzymes comprises MMP-1 (matrix metalloproteinase-1, i.e., collagenase-1), MMP-8 (i.e., collagenase-2), and MMP-13 (i.e., collagenase-3). The latter enzyme is expressed only in OA chondrocytes and possesses the highest activity against collagen 2. It represents the final hub of a variety of signals and multiple effectors that converge upon its regulation [20]. Interestingly, fine-tuning of collagenase activity is exerted through local proteolytic activation (by MMP-10 [28] or plasmin [29]) and inhibition (by means of the tissue inhibitor of matrix metalloproteinases or TIMPs). 

Based on what has been described, ADAMTS-5 and MMP-13 are the major catabolic enzymes in OA. Other enzymes exert complementary roles, such as stromelysin MMP-3, able to cleave proteoglycan, and collagen IX, the latter representing a minor cartilage ECM component found at the surface of collagen 2 fibrils particularly in the chondrons, i.e., at the level of the territorial matrix [30]. 

In most cases, inflammatory stimuli drive the loss of maturational arrest via activation of NF-κB pathway [31]. Besides the promotion of catabolic enzymes, NF-κB activation leads to increased expression of known targets, such as COX-2 and inducible nitric oxide synthase (iNOS), and downstream products, e.g., prostaglandins (such as prostaglandin E2, PGE2, involved in pain) and nitric oxide (NO).

Most of the in vitro research carried out to understand the molecular mechanisms underlying OA has employed IL-1β, which has also been confirmed to represent an OA target [19] As nicely reviewed in Reference [32], IL-1β triggers in chondrocytes a variety of signaling pathways, that include the mitogen-activated protein kinases, MAPKs (extracellular signal-regulated kinase 1 and 2, ERK1/2, p38 MAPK, and c-Jun N-terminal kinase 1 and 2, JNK1/2), which synergize with NF-κB to drive effects on chondrocyte metabolism, ECM, and inflammatory mediators. Moreover, despite the focus on cartilage and chondrocytes, OA is a whole-joint disease, as the inflammatory environment triggers many cells in the nearby tissues (bone: osteoblasts, osteocytes, and osteoclasts; synovium: synovial fibroblasts, macrophages derived from blood monocytes that are recruited to the synovium and once there switch to a M1 inflammatory phenotype, and even neutrophils and lymphocytes; infrapatellar fat pad: adipocytes), but also the adult stem cells contained in joint tissues that amplify the inflammatory–catabolic networks [32]. 

Stimuli from this environment induce inflammatory and catabolic genes both directly and via epigenetic pathways, as confirmed by findings obtained either from in vitro experiments or from analysis of OA tissues. Marked changes of the miRNome occur in OA, as reviewed in Reference [33]. Increase of the major OA catabolic enzymes, MMP-13 and ADAMTS5, is facilitated by the NF-κB-dependent reduction of miR-27b [34] and miR-140 [35], respectively. 

Moreover, changes in the methylome of OA cartilage point at a profound effect of oxidative stress in OA articular cartilage, given that miR-9 was found among the hypomethylated genes in a genome-wide analysis [36], suggesting that its gene expression may respond to the oxidative state of the cell. According to these findings, miR-9 is rapidly induced following H_2_O_2_ exposure, and reduces the expression of sirtuin 1 (SIRT-1) [37], a key metabolic regulator in chondrocytes [38]. Together with 5’ adenosine monophosphate-activated protein kinase (AMPK), SIRT-1 provides “stop signals” for oxidative stress, inflammatory, and matrix catabolic processes in chondrocytes, and is also involved in mitochondrial biogenesis [37]. An additional pathway to counteract cartilage derangement is via the promotion of autophagy [39], i.e., recycling of damaged macromolecules and organelles. To execute this key homeostatic mechanism, the damaged organelle must be segregated in a double membrane vacuole (autophagosome). At this stage, the cargo is immobilized to the inner autophagosome membrane, tagged by microtubule-associated protein light chain 3 (LC3)-II, via the autophagic adapter p62. The autophagosome is then ready to fuse with lysosome (autolysosome), so that the lysosomal enzymes may digest the cargo [40]. Increased gene expression and decreased protein expression of p62 are suggestive of efficient autophagy and autophagic flux, as its gene transcription is active to restore its intracellular levels, which are constantly decreased by degradation through the autophagic process. 

Current knowledge indicates that loss of maturational arrest in OA overlaps with oxidative stress and associated macromolecular/organelle dysfunction, leading to cell senescence and low-grade inflammation [12]. Depending on the local environment and the magnitude of oxidative stress and DNA damage, chondrocytes may proceed down different pathways of the DNA-damage response, either pro-survival or pro-elimination [41]. Among the latter, differentiation, senescence, and apoptosis are ordered according to an increasing level of DNA damage [41]. Apoptosis occurs following activation of caspase 3, mediated by the caspase-9-dependent mitochondrial pathway of apoptosis [41]. Loss of maturational arrest and progression into the default route of hypertrophy and terminal differentiation correspond to an aging of articular chondrocytes, which indeed increases expression of p16, a senescence marker. Once again, epigenetic networks are at work, since increased p16 expression is the result of inflammation-dependent miR-24 reduction [42]. The relevance of senescence in OA is supported by increasing evidence that targeting of senescent cells via senolytics might actually correspond to a disease-modifying strategy [43,44]. Senescent cells are not only dysfunctional, but also affect the functionality of bystander cells [45] through the release of cytokines (including IL-1, IL-6), chemokines (including IL-8, monocyte chemoattractant protein 1 (MCP-1), and macrophage inflammatory protein 1α (MIP-1α)), and matrix-degrading enzymes (including MMP-1, MMP-13, MMP-10, and MMP-3) that collectively constitute the senescence-associated secretory phenotype (SASP) [46]. The inflammatory environment worsens the functionality of autophagy, which is already impaired with aging [47], and the lack of turnover of the mitochondria leads to further enhancement of oxidative stress [48], i.e., an “imbalance between oxidants and antioxidants in favour of the oxidants, leading to disruption of redox signaling and control and/or molecular damage” [49].

This picture is shared by major age-associated degenerative diseases, which, according to the geroscience hypothesis [50], are the result of common and interdependent conditions that include chronic low-grade inflammation, macromolecular/organelle dysfunction, stem cell dysfunction [51], and accumulation of senescent cells. It is becoming clear that targeting one of the above-mentioned conditions has mitigating effects on the others and that “mammalian aging can be delayed with genetic, dietary, and pharmacologic approaches” [50].

From a molecular point of view, these alterations are the consequences of some dysfunction in the seven connected and intertwined “pillars of aging” (adaptation to stress, epigenetics, inflammation, macromolecular damage, metabolism, proteostasis, and stem cells and regeneration) [50].

## 3. Efficacy of Current Nutraceuticals in Osteoarthritis and Their Nutrigenomic Role

Nutritional components derived from the human diet, such as amino acids, glucids, fatty acids, vitamins, minerals, and other natural agents, can not only fulfil a structural function in the cell, but also represent molecular signals able to influence biochemical pathways. Indeed, they can directly interact with key factors and modulate the pathways implicated in pathogenesis, or influence intra- and extracellular microenvironments, thereby indirectly modifying cellular activities. Several studies have found a potential of nutraceuticals in clinical practice based on the antioxidant, anti-inflammatory and anticatabolic effects exhibited by their supplementation in OA management [52]. Interestingly, a lower prevalence of OA has been associated with adherence to the Mediterranean diet, defined by frequent consumption of cereals, fruits, vegetables, legumes, fish, and olive oil [53]. The mechanistic reasons underlying these epidemiological findings are related to OA pathogenesis: inflammation and oxidative stress are not secondary and complicating events—rather, they are involved in triggering OA onset. Thus, the intake of nutraceuticals with antioxidant and anti-inflammatory properties can represent a rational strategy in “early” OA patients. On the other hand, nutraceutical supplementation can sustain the conventional pharmacotherapy in “medium or advanced” OA patients by slowing down the disease progression. Besides the mechanisms of action described in other reviews [4,6,52], many bioactive dietary molecules have been shown to significantly modify the gene expression of OA markers, as well as other cellular components involved in homeostatic mechanisms [54]. For this reason, these molecules are being studied by the branch of nutrigenomics focused on deciphering the role of nutrients and bioactive food-derived compounds on the transcriptome, and thus on the proteome and the metabolome. An interesting subgroup includes those molecules able to even change epigenetic marks, thereby potentially able to reverse the acquired degradative phenotype of OA chondrocytes.

From a chemical point of view, most of the dietary supplements currently proposed for OA belong to the following categories: natural components of cartilage and synovial fluid, such as glucosamine, hyaluronic acid, and chondroitin sulfate; lipids and fatty acids; organosulfur compounds; and flavonoid and non-flavonoid polyphenols. Flavonoids, in particular, a group of benzo-pyrone derivatives, are widely distributed in many plants and, among different functions, their phenolic structure is responsible for filtering UV, and thus for the color and aroma of flowers. Flavonoids include several subclasses, namely flavones, flavonols, flavanones, flavanonols, flavanols or catechins, anthocyanins, and chalcones [55]. Being natural radical scavengers, besides their antioxidant activity, compounds in this family exert a known anti-inflammatory function, in particular against the enzyme COX-2 [56], the target of most NSAIDs. Given the wide employment of this class of drugs in the therapy of OA patients and the related side effects, flavonoids are of special interest for researchers and rheumatologists in the development of a combined therapeutic strategy targeted to reduce NSAID doses and toxicity.

**Figure 1:**
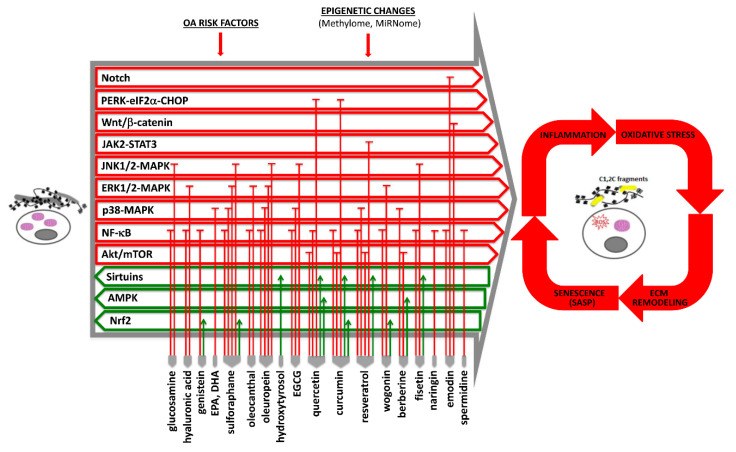
During osteoarthritis (OA) onset and progression, chondrocytes lose their healthy, differentiation-arrested phenotype (represented on the left) and enter hypertrophy and terminal differentiation (represented on the right). This occurs under the effect of different OA risk factors (ageing, mechanical stress, obesity, genetic alteration, and inflammation). Despite the different initial triggers, cartilage with established OA features shows similar patterns of signaling pathways, transcription factors, and epigenetic changes. The final outcome is a tissue in which the cells are exposed to a vicious cycle of inflammatory signals and oxidative stress that boost each other and also sustain extracellular matrix (ECM) remodeling. The increased ECM catabolism further worsens the senescence of articular chondrocytes because they are able to release high levels of senescence-associated secretory phenotype (SASP) molecules that further trigger inflammation. These pathogenetic mechanisms may be targeted by nutraceuticals that are able to both counteract pro-inflammatory and catabolic pathways and enhance the activity of homeostatic mechanisms, according to currently available evidence reported in this review. Abbreviations: AMPK, 5’ adenosine monophosphate-activated protein kinase; CHOP, C/EBP homologous protein; DHA, docosahexaenoic acid; EGCG, epigallocatechin-3-gallate; eIF2α, α-subunit of eukaryotic translation initiation factor 2; EPA, eicosapentaenoic acid; ERK, extracellular signal-regulated kinase 1 and 2; JAK2/STAT3, Janus kinase 2/signal transducer and activator of transcription 3; JNK1/2, c-Jun N-terminal kinase 1 and 2; MAPK, mitogen-activated protein kinase; mTOR, mammalian target of rapamycin; NF-κB, nuclear factor kappa-light-chain-enhancer of activated B cells; Nrf2, nuclear factor erythroid 2-related factor 2; PERK, protein kinase-like endoplasmic reticulum kinase.

Current available evidence of the effects of nutraceuticals in OA pathophysiology, with mention of the affected signaling pathways and homeostatic mechanisms, is outlined in Figure 1 (and further detailed in Table S1).

### 3.1. Glucosamine, Chondroitin Sulfate, and Hyaluronic Acid

The most common nutraceuticals employed as dietary supplements in OA therapy are glucosamine (GlcN), or 2-amino-2-deoxy-d-glucose (C_6_H_13_NO_5_), and chondroitin sulfate (CS). The first one, isolated from the chitin of the exoskeleton of crustaceans and from mushrooms, is an amino monosaccharide and component of glycosaminoglycan (GAG) chains. The latter is a GAG composed of the alternating sugars d-glucuronic acid and *N*-acetyl-d-galactosamine sulfate. Single treatments or a combination of both have shown to relief symptoms and improve tissue structure [57], and are currently prescribed in clinical practice. Furthermore, these compounds, in single or combined treatment, have been demonstrated to reduce IL-1β-induced mRNA expression of MMP-3, MMP-13, aggrecanase-1, aggrecanase-2, and JNK [57,58,59].

Interestingly, Imagawa et al. investigated the potential of GlcN to modulate NF-κB activity and cytokine-induced gene expression in articular chondrocytes via influencing epigenetic mechanisms [60]. Although a single treatment of GlcN and NF-κB inhibitor (BAY 11-7082) led to no effect on the methylation status of the IL-1β promoter, the methylation levels of CpG islands in the IL-1β promoter were reversed by GlcN and BAY 11-7082 to 44% and 53%, respectively, in combination with cytokine treatments (IL-1β and oncostatin M) [60]. These data indicate that GlcN represses, possibly via NF-κB inhibition, the pro-inflammatory signals by making the IL-1β gene less prone to being transcribed. 

Another important natural component of synovial fluid is hyaluronic acid. This molecule contributes to joint fluid viscosity and elasticity; thus, its supplementation either intra-articularly or orally [61] can reduce pain via lubrication and increasing viscoelastic properties. An extensive review of hyaluronic acid mechanisms of action in OA is available in Reference [62]. The effects include the increase of articular cartilage lubrication, antioxidative/antinitrosative, analgesic, anti-inflammatory and chondroprotective effects, the prevention of ECM degradation, and the promotion of cartilage repair. According to Yatabe [63], hyaluronic acid is effective in reducing ADAMTS-4 via a combined inhibition of NF-κB and ERK1/2 pathways. 

### 3.2. Avocado/Soybean Unsaponifiables

Among the symptomatic slow-acting drugs for OA treatment (SYSAD-OA), avocado/soybean unsaponifiables (ASU), as well as GlcN and CS, have been shown to be effective in relieving some symptoms. ASU include the total fraction of unsaponifiables of avocado and soybean oils in a proportion of one to two thirds. The chondroprotective effect of ASU could be related to the ability to enhance transforming growth factor-β (TGF-β) expression in these cells. Boumediene and colleagues identified the cis-acting sequences mediating ASU responsiveness in the TGF-β1 promoters by using different promoter constructs. Treatment of bovine articular chondrocytes transfected with these constructs suggested that the effect on TGF-β1 expression is mediated by the region located between −732 and −1132 bp. Further, ASU increased the production of plasminogen activator inhibitor (PAI)-1, thus blocking the plasmin cascade that is responsible for metalloprotease activation [64]. Another investigated aspect concerns the effects of ASU treatment on chondrocyte metabolism mediated by osteoblasts in a co-culture system. The authors concluded that ASU counteracts the inhibition of matrix molecule synthesis exerted by OA osteoblasts, thereby significantly rescuing type II collagen gene expression [65].

Belonging to the isoflavone category, genistein is naturally occurring in soy products, and thus in ASU too, and it is known to exert estrogen-like activity despite its non-steroidal structure. An initial study providing evidence of the possible beneficial effect of dietary supplementation of soy-derived compounds in OA management was published in 2004 [66] and showed decreased markers and symptoms of OA after three months, more evident in men than in women. Afterwards, several studies investigated possible mechanisms of action of genistein and other phytoestrogens, finding in different conditions and experimental models the reduced production of mediators of inflammation, like COX-2, NO [67], and iNOS, and of cartilage degradation, like MMPs [68], thus pointing at the tuning of the NF-κB pathway [69]. Moreover, it has also been demonstrated that genistein can exert a protective action on chondrocytes by improving the DNA binding of the nuclear factor erythroid 2-related factor 2 (Nrf2), a major regulator of cellular response to oxidative stress. Among its downstream genes there is the gene coding for heme oxygenase-1 (HO-1), by which Nrf2 may control NF-κB activation [68].

### 3.3. Omega-3 Polyunsaturated Fatty Acids

Omega-3 fatty acids are classified as long-chain polyunsaturated fatty acids (LC-PUFAs) that include alpha-linolenic (ALA, 18:3 ω-3), eicosapentaenoic (EPA, 20:5 ω-3), and docosahexaenoic (DHA, 22:6 ω-3) acids. They may incorporate into cell membranes contributing to both fluidity and permeability of the plasma and mitochondrial bilayers. ALA cannot be synthesized by humans and, as such, is considered an essential fatty acid (EFA). Although EPA and DHA can be synthesized from the ALA precursor through the enzymatic action of elongases, the conversion ratio is so low (especially for DHA) that they can also be considered “conditionally essential” components of our diet. Indeed, EPA and DHA supplementation is recommended in several conditions, particularly during fetal development or in cardiovascular disease, as well as in degenerative diseases associated with chronic inflammation, such as brain disorders [70]. The anticatabolic activity of these fatty acids against matrix degradation in OA has been related to the capability to modulate gene expression of key proteins involved in proteolysis and inflammation. In 2009, Zainal et al. reported that ALA, EPA, and DHA can reduce the mRNA levels of ADAMTS-4, ADAMTS-5, MMP-3, MMP-13, COX-2 (but not COX-1), IL-1α, IL-1β, and tumor necrosis factor α (TNF-α) in IL-1α-treated chondrocyte cultures. They compared the relative efficacy of the three fatty acids and their studies clearly showed that all of them are active, with EPA being the most effective, followed by DHA and then by ALA [71]. Later, Sakata and colleagues showed that intra-articular injection of EPA protected chondrocytes from oxidative-stress-induced apoptosis and reduced MMP-13 expression in a mouse model of OA. Furthermore, the parallel in vitro study demonstrated that the extent of sodium-nitroprusside-induced apoptosis and related markers (caspase-3 and poly(ADP-ribose) polymerase cleavage, phosphorylation of p38 MAPK and p53) was reduced when chondrocytes were pretreated with EPA [72]. The role of DHA in inhibiting cartilage degeneration in OA was assessed in both IL-1β-stimulated human chondrosarcoma SW1353 cells and in a rat model of adjuvant-induced arthritis (AIA). In particular, the authors clarified the mechanism underlying DHA effect on MMP-13 expression; among the group of serine/threonine protein kinases, DHA was able to selectively block IL-1β-induced p38 activation, rather than the activation of either JNK or ERK [73]. 

### 3.4. Sulforaphane and Organosulfur Compounds

Mainly present as a glucosinolate precursor (glucoraphanin) in cruciferous vegetables such as broccoli, sulforaphane is an isothiocyanate obtained after myrosinase activation. The first in vitro evidence of a potential benefit of sulforaphane in OA was reported in 2011, when it was shown that this compound is able to protect chondrocytes against oxidative stress and apoptosis induced by inflammatory cytokines and chemokines [74]. Further investigations analyzed in vitro and in vivo the molecular mechanisms underlying this protective effect, demonstrating the downregulation of pro-inflammatory and joint degeneration markers such as COX-2, ADAMTS-5, and MMP-2 [75]. This suppressed cartilage destruction seems to be related to the repression of NF-κB signaling by preventing its DNA binding and thus the transcription of NF-κB-dependent genes [76]. In addition to the evidence of the effects directly exerted on chondrocytes, sulforaphane has proven to be a compound with immunomodulatory activity on monocytes [77], suggesting multiple mechanisms of action by which it provides a benefit in OA, including the tuning of innate immunity. A first proof-of-principle study on OA patients showed the effective feasibility of a therapeutic intervention based on supplementation with foods containing high doses of glucosinolates; in fact, their derived isothiocyanates were detectable in plasma as well as in synovial fluid and were able to induce a modification in its proteome [78]. These findings support the high bioavailability of these nutraceuticals, which allows them to exert multiple activities at both local and systemic levels, thus attenuating local and systemic inflammation.

In a recent study, the chondroprotective ability of allicin, another food-derived organosulfur compound mainly present in garlic, was compared to that of sulforaphane and lycopene (a carotenoid with eleven conjugated double bonds), showing a similar antioxidative potential [79]. 

### 3.5. Olive-Derived Compounds

Olive oil, a basic component of the Mediterranean diet, and its derivatives, in particular bioactive polyphenolic compounds (oleocanthal (OC), oleuropein (OP), tyrosol (TY), and hydroxytyrosol (HT)), are of particular interest for the management of many inflammatory and degenerative diseases, including OA. Previous reviews have already described their properties [54,80], including in the context of OA, but without focusing on the nutrigenomic role of these promising nutraceuticals, which has only recently emerged. From the point of view of composition, olive oil can be divided into several fractions. The major one includes the saponifiable fraction, mostly triglycerides, mainly represented by oleic acid (18:1 n-9) and with lower amounts of palmitic, stearic, linoleic, and α-linoleic acids. The minor fraction comprises both the unsaponifiable components and the soluble part, which contains the phenolic compounds. The latter are further classified into two subgroups: lipophilic phenols (tocopherols and tocotrienols) and hydrophilic phenols (phenolic acids, phenolic alcohols, lignans, and flavonoids). OC, OP, and HT belong to the second group and are responsible for most of the beneficial effects on human health ascribed to olive oil [81].

Gualillo and colleagues demonstrated that OC and a related synthetic derivative decreased lipopolysaccharide (LPS)-induced iNOS expression and NO production in the ATDC5 murine chondrogenic cell line [82]. Later, the same authors reported the OC-mediated inhibition of the pro-inflammatory mediators MIP-1α and IL-6 in both ATDC5 chondrocytes and J774 macrophages. The ability to attenuate inflammatory networks not only in chondrocytes but also in macrophages is of interest, since macrophages can be recruited to synovium by inflammatory mediators released by chondrocytes, and the crosstalk between these two cell types contributes to the chronic joint inflammation found in the disease [83]. The authors further extended the study of OC effects in human primary chondrocytes and demonstrated that OC inhibits TNF-α, IL-8, and IL-6 mRNA expression and MMP-13 and ADAMTS-5 protein expression. To investigate the upstream mechanisms underlying OC-induced gene modulation, they focused their attention on the NF-κB pathway. Indeed, OC is able to increase IκB, responsible for the NF-κB sequestration in the cytosol, thus reducing the nuclear amount of p65-NF-κB. Nuclear p65 exerts a key role in the induction of gene expression of inflammatory cytokines and mediators (IL-6, IL-8, COX-2, iNOS, MIP-1α, TNF-α, LCN2) and catabolic enzymes (MMP13 and ADAMTS-5) [84].

The other phenolic components of olive oil have also shown interesting anti-inflammatory properties in OA. Indeed, Feng et al. reported that OP significantly inhibits the IL-1β-induced production of NO and PGE2, the gene expression of COX-2, iNOS, MMP-1, MMP-13, and ADAMTS-5, and therefore the degradation of aggrecan and collagen-II in human primary chondrocytes. This beneficial effect has been associated with the repression of NF-κB and MAPK pathways [85].

Besides being a known antioxidant agent, HT, derived from the hydrolysis of OP, has shown peculiar activities in counteracting OA pathogenesis. Indeed, HT is able to reduce oxidative-stress-induced apoptosis, inflammation, catabolism, chondrocyte terminal differentiation, and angiogenesis, as indicated by the decrease of caspase-3 activity, gene expression of COX-2 and iNOS, MMP-13, RUNX-2, and vascular endothelial growth factor (VEGF), respectively [86]. Furthermore, findings from several groups have shown that HT exerts its chondroprotective activity by stimulating the flux of autophagy, a housekeeping cellular system able to remove and recycle damaged macromolecules and organelles [87,88]. This process has been reported to decrease in several age-related diseases, including OA [47]; therefore, a molecule able to restore its function represents a promising agent for the adjuvant therapy of OA. By using functional genomics, the mechanisms underlying the effects of HT were revealed. The inhibition of autophagy partially reversed the cytoprotective effect of HT against H_2_O_2_-induced oxidative stress, indicating the role of autophagy in HT chondroprotection. Moreover, it has been shown that the autophagy-promoting activity is driven by the deacetylase SIRT-1, implicated in cell survival and cartilage homeostasis. The silencing of this enzyme indeed prevented HT-mediated chondroprotection [87]. The nutrigenomic role of HT in OA emerged via its demonstrated effects on gene expression, in particular of sequestosome 1 (SQSTM1/p62), required for the autophagic degradation of polyubiquitin-containing bodies [87], and also of microRNA (miR)-9 [37]. The latter belongs to the large family of small non-coding RNAs, potent post-transcriptional regulators able to limit the expression of specific mRNA targets through their direct interaction followed by degradation. HT has been shown to counteract H_2_O_2_-induced miR-9 expression, thereby restoring the protein translation of its cognate target, SIRT-1 [37]. In humans, three independent genomic loci of miR-9 map to chromosomes 1q22 (MIR9-1), 5q14.3 (MIR9-2), and 15q26.1 (MIR9-3), and HT prevents the promoter demethylation of these three genes occurring in oxidative stress conditions [89]. This recent paper ascribed to HT an interesting role as an epigenetic modulator able to influence gene expression without changing the genetic profile. Furthermore, in line with these papers, Zhi et al. reported that HT promoted autophagy flux by increasing the gene expression of SIRT-6 in chondrocytes stimulated with TNF-α [88].

### 3.6. Green Tea Polyphenols

Consuming green tea has been associated with many health benefits, including anti-inflammatory, antioxidant, and antimicrobial [90] effects, mainly ascribed to the content in polyphenols. Most of the green tea polyphenols are flavonols, commonly known as catechins (epicatechin, epigallocatechin, epicatechin-3-gallate, and epigallocatechin-3-gallate (EGCG)). Besides the antioxidant activity of ECGC, the most studied bioactive derivative of green tea, many findings also describe its antiphlogistic ability. Indeed, in vitro studies showed that prior to IL-1β delivery, pretreatment of human chondrocytes with EGCG resulted in a dose-dependent inhibition of the production of NO and PGE2, correlating with both the inhibition of iNOS and COX-2 gene expression and a reduced catalytic activity of the two enzymes [91]. In parallel, Singh et al. reported that EGCG inhibits NO production and iNOS expression in human chondrocytes by suppressing the degradation of inhibitor of NF-κB α (IκBα), and thus by retaining NF-κB in the cytoplasm [92]. Furthermore, EGCG proved to have an anticatabolic activity by decreasing the gene expression and production of MMP-13 stimulated by AGEs, as well as of TNFα in human chondrocytes through the suppression of p38-MAPK, JNK, and NF-κB activation [93]. Interestingly, green tea polyphenols proved as promising epigenome modulators by regulating two miRs, miR-199a-3p [94], and miR-9 [95]. The role of miR-199-3p has been highlighted in the EGCG-mediated reduction of COX-2 mRNA expression and of prostaglandin E2 (PGE2) production in human chondrocytes stimulated with IL-1β. These data were confirmed by assessing the effect of transfection of either a miR mimic or an inhibitor [94]. Recently, Zhang et al. demonstrated that pretreatment with these compounds can reduce LPS-induced inflammatory cell damage in the ATDC5 cell line by suppressing the MAPK and NF-κB pathways through miR-9 regulation [95]. A mouse DMM model was employed to test the potential of EGCG in relieving OA-associated cartilage damage and pain. The administration of EGCG (25 mg/kg) or control vehicle was executed via intraperitoneal injection. Four and eight weeks after DMM surgery and EGCG treatment, EGCG-treated mice showed less proteoglycan loss in the articular cartilage, visualized by Safranin O staining, less cartilage erosion, and reduction of MMP-13 and ADAMTS5 staining compared to control mice. Moreover, EGCG decreased the levels of MMP1, MMP3, MMP8, MMP13, ADAMTS5, IL-1β, and TNFα mRNAs [96].

### 3.7. Quercetin

Quercetin, the circulating aglycone form of rutin, is a flavonol widely present in vegetables. With a polyphenolic structure, this compound has a strong reactive oxygen species (ROS) scavenging power [97] and it has been also classified as a phytoestrogen [98]. Moreover, rutosides are known to influence the metabolism and crosslinking of collagen [99]. Quercetin is also a component of one of the most used Chinese herbal medicines in OA patients, *Achyranthes bidentate*. Several studies have investigated the therapeutic actions of this compound in in vivo models of OA, finding beneficial effects of both topical application [100,101] and oral supplementation [102,103]. More recently, the mechanism of action for this polyphenol was elucidated: the promotion of mitochondrial biogenesis and efficiency by increasing membrane potential, oxygen consumption, ATP levels, glutathione and glutathione peroxidase levels, and decreasing oxidative stress that may affect mitochondrial functioning. These effects seem to be mediated by the induction of the phosphorylated status of AMPK and the parallel increased expression of SIRT-1. The latter may enhance mitochondrial efficiency by inducing the expression of mitochondrial biogenesis genes such as mitochondrial transcription factor A (TFAM), nuclear respiratory factors 1 and 2, and their master regulator PGC-1α (peroxisome proliferator-activated receptor ϒ co-activator 1 α), which is active in the deacetylated form (possibly mediated by SIRT-1) [104]. Moreover, other authors demonstrated that the activation of SIRT-1 and AMPK also attenuates the endoplasmic reticulum (ER) stress in rat chondrocytes and in an OA rat model, thus improving cartilage maintenance and chondrocyte survival [105]. In combination with these mechanisms of action, it was also demonstrated that quercetin has an impact on the polarization of synovial macrophages to M2 macrophages [106], thus reducing the accumulation of synovial M1 macrophages that sustain OA exacerbation in both human and mice. Moreover, quercetin is able to increase the expression of TGF-β and insulin-like growth factor (IGF), which promote a prochondrogenic microenvironment and the synthesis of GAG in chondrocytes, thus improving the cartilage’s ability to repair [107]. Collectively, these data from animal models strongly support further studies addressed to the use of quercetin as a supplement for human OA treatment.

### 3.8. Curcumin

Another compound that is drawing increasing attention for its possible benefits in OA treatment is curcumin. This polyphenol, also known as diferuloylmethane, is the main biochemical component extracted from the roots of turmeric (*Curcuma longa*). Known for thousands of years for its anti-inflammatory properties, turmeric has long been used in traditional Oriental medicine for the treatment of a wide range of pathologies including OA. Turmeric is mostly used as a spice in the rest of the world but, in the last few decades, interest around it has grown in the scientific community due to its anti-inflammatory, antioxidant, and anticatabolic properties [108]. Many studies support the involvement of the NF-κB signaling pathway in curcumin’s anti-inflammatory properties [109,110,111]. Through this interaction, curcumin is able to reduce the expression of inflammatory and OA markers such as IL-1β, TNF-α, iNOS, COX-2, and MMPs, especially MMP-13, which are among the major factors responsible for the inflammation and extracellular matrix degradation involved in OA onset and progression. A possible mechanism of action was revealed in an in vitro IL-1-induced OA model, where curcumin proved to be able to upregulate type II collagen production and reduce MMP13 expression. Both effects were due to the blockade of IκBα phosphorylation, thus preventing NF-κB p65/RelA subunit translocation in the nucleus [110]. The signaling axis represented by ROS, Nrf2, and its target genes such as HO-1, superoxide dismutase 2 (SOD2), NAD(P)H: quinone oxidoreductase (NQO1) and glutamate-cysteine ligase catalytic subunit, also seems to exert an important role in mediating the anti-inflammatory and antioxidant effects of curcumin, as shown in in vitro and in vivo models of temporomandibular joint osteoarthritis (TMJ OA), a subtype of OA [112].

Moreover, curcumin exerts further upstream control over NF-κB, inhibiting the pathway of toll-like receptor 4 (TLR4), able to mediate the sterile inflammatory response involved in OA pathogenesis [111]. Inhibition of NF-κB by curcumin also creates dose-dependent protection from accumulation of AGEs and consequent upregulation of TNF-α and MMP-13, a known mechanism of OA progression [109]. Oxidative-stress-induced endoplasmic reticulum (ER) stress is another feature displayed in OA. Feng and colleagues demonstrated that curcumin can reduce oxidative and ER stress in a surgically induced OA animal model by increasing the expression of the deacetylase SIRT-1, which in turn reduced the activity of the phospho-PERK (protein kinase-like endoplasmic reticulum kinase), Phospho-eIF2α (α-subunit of eukaryotic translation initiation factor 2), and CHOP (C/EBP homologous protein) axis, activated by cellular stress. Curcumin-induced upregulation of SIRT-1 led to a reduction of ER stress, diminished apoptosis, and protected chondrocytes from further progression of OA [113]. Finally, curcumin is involved in the regulation of other pivotal homeostasis mechanisms such as autophagy. For the first time, Zhang and colleagues proved that curcumin exhibits a strong effect in promoting autophagy in both a spontaneous and a DMM-induced OA model. Increased autophagy, by attenuation of the Akt/mTOR (mammalian target of rapamycin) pathway, is beneficial in OA, since it leads to decreased apoptosis and reduced matrix degradation [114].

In spite of the growing evidence pointing at a potential therapeutic role of curcumin in OA, a few major issues remain unresolved. The bioavailability of this phytochemical compound is very low (below 1%) and so is its solubility, especially through oral consumption [111]. Several studies have tried to overcome this limitation. Thus far, the most successful approaches have consisted of intra-articular injections [111] or the creation of nanoparticle- or liposome-based curcumin formulations, but efficiency in systemic delivery is far from being achieved [115]. An interesting perspective comes from the development of combination of curcumin and other nutraceuticals, each used at a lower dose than would be used for a single compound, in order to exploit synergistic therapeutic effects. This strategy allows the beneficial effects to be maximized while minimizing the toxicity of higher doses or the resistance to the treatment. In a recent study, the combination of curcumin and two other compounds, flavocoxid and β-caryophyllene, indeed proved to be more effective in reducing LPS- and IL-1β-induced inflammation in a synergistic fashion compared to the natural products singularly. The combination also stimulated the expression of collagen II [115]. Another strategy for improving systemic delivery could be solubility enhancement by forming a complex with compounds with known affinity for polyphenolics. In a recent study, such a complex was formed between curcumin and soy lecithin. The complex, with its enhanced bioavailability, showed the consistent anti-inflammatory and chondroprotective properties typical of curcumin alone, along with reduction of LPS-induced inflammatory markers such as TNF-α, IL-6, IL-8, iNOS, and COX-2 [116].

### 3.9. Resveratrol

Resveratrol (trans-3,5,4’-trihydroxystilbene) is a phenolic compound present in a large number of plants and fruits including grape skin, wines, mulberries, and peanuts. It belongs to a group of natural compounds known as phytoalexins that exert a pivotal role in protecting plants from microbial infections. This task requires anti-inflammatory, immunomodulatory, and anti-apoptotic properties [117,118]. These properties explain the initial interest around this compound for its potential use as therapeutic agent against cancer [119]. Later, a possible application in OA came from early studies which confirmed that resveratrol has a chondroprotective activity in limiting cartilage matrix loss during the early stages of the disease [120], and an anti-apoptotic activity through reduction of the IL-1β/caspase-3/PARP axis and increase of p53 degradation [117,121]. Subsequent studies confirmed these results and implicated several mechanisms. Resveratrol inhibition of IL-1β-mediated apoptosis can occur by both blocking NF-κB and its target gene products [122] and through inhibition of COX-2-dependent PGE2 release which impacts mitochondrial membrane depolarization and function [123]. The chondroprotective activity of resveratrol was also confirmed by Liu and colleagues, who showed that resveratrol might be responsible for both anti-inflammatory effects (reduction of NF-κB-dependent genes: iNOS, COX-2, and PGE2) and anticatabolic activity in a model of pig articular chondrocytes stimulated by AGEs [124]. Furthermore, SIRT-1-mediated activity seems to modulate NF-κB after treatment with resveratrol in rat cartilage [125]. SIRT-1/NF-κB involvement in chondroprotection, with the additional interaction of hypoxia-inducible factor 2α (HIF-2α), was confirmed in vivo in a mouse DMM model through intra-articular injection of resveratrol [126]. Gu and colleagues demonstrated that the IL-1β modulation exerted by resveratrol is both TLR4/MyD88-dependent and –independent [127,128]. Interesting results also came from studies focused on a particular subtype of OA, the obesity-related one. In C57BL/6 mice prone to developing OA and subjected to a high-fat diet, oral treatment with resveratrol showed benefits in slowing down the progression of the pathology in comparison to a control group [129,130]. In the same model, a delay of cartilage degradation was also linked to a resveratrol-induced activation of autophagy [131], and a protective role was attributed to the modulation of the JAK2/STAT3 (Janus kinase 2/signal transducer and activator of transcription 3) pathway [132]. In a recent study, Jin and colleagues addressed the increasingly significant topic of miRNA by linking resveratrol treatment with a chondroprotective effect exerted by the regulation of miR-146 in a LPS-induced OA model in vitro [133]. In contrast to other natural compounds, resveratrol is available as a dietary supplement and its safety has long been proven. Its efficacy in ameliorating OA symptoms was shown in two clinical studies in combination with other NSAIDs commonly used for OA such as meloxicam [134,135].

Taken together, all these studies provide a wide range of evidence of resveratrol-mediated biological activities with potential beneficial outcomes in OA.

### 3.10. Wogonin

Recently, there has been increased interest in the potential use of wogonin in OA. This O-methylated flavone is present in *Scutellaria baicalensis*, an herb used in traditional Chinese medicine. Wogonin, already known for its anti-inflammatory and anticarcinogenic effects, has demonstrated ability to reduce the expression of MMP-3 after IL-1β treatment on articular chondrocytes [136]. Moreover, IL-1β-induced activator protein-1 (AP-1), an important transcription factor controlling the expression of cytokines, chemokines, and MMPs, is suppressed by wogonin [137]. The same authors further elucidated its mechanism of action and found that wogonin is able to induce low levels of ROS in OA chondrocytes, thus inducing Nrf2 and enzymes involved in antioxidant-response pathways like HO-1, SOD2, and NQO1 [138]. An interesting paper also investigated the ability of this molecule to directly interact with the DNA of chondrocytes, since it was found that it mainly localizes in the cell nucleus. In particular, an in silico molecular docking experiment unveiled the potential mode of interaction with DNA: wogonin intercalates between guanine and cytosine bases, as ethidium bromide. Moreover, it seems that wogonin prevents DNA denaturation, thus contributing to genomic DNA stability [139]. Considering this evidence, the topical application of this nutraceutical was very recently tested in a mouse DMM model of knee OA [140]. The results showed that a modest dose of wogonin in a topical cream improved joint health as shown by significantly decreased pathological scores (cyst-like lesions (CLLs), Osteoarthritis Research Society International (OARSI), and Mankin scores). This effect may be mediated by MMP-13, NF-κB, and high temperature requirement A serine peptidase 1 (HTRA1) decrease.

### 3.11. Berberine 

Berberine chloride (BBR, 5,6-dihydro-9,10-dimethoxybenzo[g]-1,3-benzodioxolo[5,6-a] quinolizinium chloride) is a botanical-derived isoquinoline alkaloid that can be isolated from the root and bark of different plants, including *Coptis chinensis*, *Berberis aristate*, *Rhizoma coptidis* (Huanglian), *Hydrastis Canadensis* (goldenseal), and *Cortex phellodendri* (Huangbai) [141,142]. Due to its anti-inflammatory and antioxidant properties, it has long being used in traditional Chinese medicine and is the active component of a Korean remedy known as BackJeolYuSin-tang (BYT) [143]. Early studies proved that in both a rabbit and a rat OA model, berberine can downregulate, in a dose-dependent fashion, the expression of OA markers following stimulation with IL-1β, such as MMP-1, MMP-3, MMP-13, ADAMTS-4, ADAMTS-5, COX-2. Moreover, berberine inhibited NO production and upregulated TIMP-1 while also reducing type II collagen and GAG release into the culture medium [143,144]. These results confirmed the anticatabolic and anti-inflammatory potential of berberine, suggesting its use in OA treatment. Subsequent research shed light on the possible mechanisms behind the chondroprotective role of this alkaloid. Zhao and colleagues suggested the activation of the Akt/p70S6K/S6 pathway, which is known to be involved in chondrocyte survival, exerted by berberine as the reason for its beneficial effects in cartilage damage and chondrocyte survival [145]. Furthermore, in a model of sodium nitroprusside (SNP, a source of NO)-induced chondrocyte apoptosis, the anti-apoptotic activity of berberine was also evidenced and was linked to the activation of AMPK and contemporary inhibition of the p38/MAPK signaling pathway [146]. The same research group later investigated the role of berberine in proliferation. Their data once again confirmed the beneficial role of berberine, which proved able to rescue SNP-inhibited chondrocyte proliferation via activation of the Wnt/β-catenin pathway [147]. Liu and colleagues confirmed the data previously reported, and further investigated the involvement of these pathways in a CIOA (collagenase-induced OA) animal model. They proposed that connective tissue growth factor (CCN2) is also responsible for IL-1β-induced cartilage damage. Berberine was able to modulate CCN2 activity in a dose- and time-dependent manner, preventing cartilage damage [148]. Furthermore, PI3-kinase/Akt and p38 pathways were found to be involved in berberine regulation of cytoskeletal reorganization and dedifferentiation in rabbit articular chondrocytes, ameliorating OA progression [149]. Recently, berberine was also proven to exert important anticatabolic and anti-inflammatory properties by suppressing IL-1-induced inflammation, once again through the inhibition of MAPK signaling pathway [150]. As with other active plant compounds, berberine shows issues regarding its bioavailability. However, an innovative solution was proposed with intra-articular injection of a nanoparticle that combined berberine and chitosan to maximize the biological activity, effectively contributing to the amelioration of OA in rats [151].

### 3.12. Other Emerging Compounds

The range of available nutraceutical supplementation or therapeutics in OA is constantly being enriched with new compounds, thus validating the great attention paid to this strategy.

A new promising nutraceutical is fisetin, a flavonoid naturally occurring in several fruits and vegetables, such as apples, strawberries, persimmons, onions, cucumbers, and many others. In keeping with evidence of its antioxidant and anti-inflammatory activity in many experimental models [152,153], a first study in 2017 investigated the effects of fisetin on IL-1β-stimulated human chondrocytes and in a mouse model of OA [154]. The in vitro data showed that this compound exerted an anti-inflammatory effect by opposing the increased production of NO, PGE2, TNF-α, IL-6, iNOS, COX-2, MMP-3, MMP-13, and ADAMTS-5 after IL-1β treatment. Moreover, it counteracted the degradation of Sox-9, aggrecan, and collagen-II. The mechanism underlying fisetin-mediated chondroprotection seems to be related to the induction of SIRT-1 activity. In vivo data from mice confirmed the protective activity of this compound on cartilage, attenuating the progression of OA [154]. 

Several papers have shown that citrus fruits are good sources of flavonoids. Among these compounds, naringin has been demonstrated to be potentially beneficial in OA. In particular, oral administration of naringin to surgically induced OA mice protected against cartilage degradation and reduced markers of OA. These effects were mediated by the suppression of NF-κB signal [155]. Moreover, another study confirmed the chondroprotective ability of naringin in a different OA model, monosodium-iodoacetate-induced OA rats [156], with reduced levels of effectors and mediators that are known to be induced by NF-κB.

An interesting compound present in several plants, including rhubarb, is emodin. This anthraquinone exerts a nutrigenomic activity by promoting the expression of the long non-coding RNA (lncRNA) Taurine up-regulated gene 1 (TUG1) in murine chondrogenic ATDC5 cells. This lncRNA may in turn counteract the activation of NF-κB and Notch pathways induced by LPS [157]. Consistently with these results, intra-articular emodin injection protected against OA progression in a rat OA model [158].

Well known as an endogenous molecule, spermidine is in all respects a nutraceutical, since its presence in some foods makes supplementation possible through diet. The first evidence of its protective potential in OA came from a study by Sacitharan and colleagues [159]. They showed that spermidine is capable of sustaining autophagy, preventing its aging-related impairment. Subsequently, our recent work showed that the protective effects of this compound on chondrocytes can be related to the inhibition of NF-κB and the reduction of DNA damage. This study also confirmed an important role of autophagy in chondroprotection. In fact, the inhibition of this process by gene silencing of ATG5 (necessary for the execution of the autophagic steps) compromised the ability of spermidine to exert its protective effects [160].

## 4. Conclusions and Perspectives

A growing body of evidence collected in animal or cellular OA models shows that nutraceutical supplementation may represent a significant adjuvant strategy in the management of OA patients, with or without the synergistic integration of conventional therapy, that could eventually be delivered at lower doses, thus reducing side effects. From a molecular perspective, most of the nutraceuticals discussed are able to intervene in the signaling pathways underlying OA pathogenesis, which combines inflammation and oxidative stress, with the former triggering the latter and vice versa. In order to increase the effectiveness of smoothing both inflammatory and oxidative mechanisms, the combined use of multiple nutraceuticals can be proposed to exploit their potential synergism. From this perspective, a recent study provided detailed molecular evidence supporting this synergism [161], and this presents a direction for future in vitro/in vivo studies. At the same time, most of these nutraceuticals were able to promote the rescue of homeostatic mechanisms, thus yielding cytoprotective and geroprotective effects. 

Although a detailed and complete knowledge of all the molecular effects is still lacking, the advantage of most of these molecules is their ability to exert pleiotropic and complementary effects ultimately targeting both inflammation and oxidative stress, as described for curcumin, while most conventional anti-inflammatory or antioxidant drugs target only one or a few of the mosaic tiles.

State-of-the-art biotechnology science is at present focused on improving the bioavailability of some of the more promising compounds.

Although the results are not yet available, based on the convincing results of the preclinical studies, some clinical trials have been issued in the last years, and can be found at https://clinicaltrials.gov/ct2/results?term=Osteoarthritis+and+nutraceutical&Search=Search. Some of these studies include molecules discussed in this review: glucosamine, hyaluronic acid, ASU, omega-3, nutraceuticals derived from grape seeds or oil, and turmeric.

Looking forward to the results of these and future studies, the perspective is to integrate the best of conventional and complementary medicine, hopefully moving towards the development of a true “disease-modifying therapy” for OA.

The data reported in the review are mostly preclinical and in vitro and, to our knowledge, are undisputed, possibly because the extent of variability is narrower in experimental compared to clinical studies. However, preclinical studies are preparatory to clinical testing, and at that stage careful implementation of principles of clinical research (such as careful selection and stratification of the patients with inclusion and exclusion criteria, power analysis, and avoidance of confounding variables) must be ensured to avoid inconsistent results.

## Supplementary Materials

The following is available online at https://www.mdpi.com/2073-4409/9/5/1232/s1, Table S1: Nutraceuticals in OA.
