# Supplementary material for: Nutraceutical Activity in Osteoarthritis Biology: A Focus on the Nutrigenomic Role
Source: Cells. 2020 May 16;9(5):1232. doi: 10.3390/cells9051232 (PMC7291002; doi:10.3390/cells9051232)
Supplement: Supplementary file 1 [file cells-09-01232-s001.pdf]

Table S1. Nutraceuticals in osteoarthritis (OA).Abbreviations: ACLT, anterior cruciate ligament transection; ADAMTS, A Disintegrin and Metalloproteinase with Thrombospondin motifs; AGE, advanced glycation end-product; AIA, adjuvant-induced arthritis; AMPK, 5' adenosine monophosphate-activated protein kinase; AP-1, activator protein 1; ASU, avocado/soybean unsaponifiables; CCN2, connective tissue growth factor; CHOP, C/EBP homologous protein; CIOA, collagenase-induced OA; COX-2, cyclooxygenase 2; CS, chondroitin sulfate; DHA, docosahexaenoic acid; DMM, destabilization of the medial meniscus; EGCG, epigallocatechin-3-gallate; eIF2α, α-subunit of eukaryotic translation initiation factor 2; EPA, eicosapentaenoic acid; ER, endoplasmic reticulum; ERK1/2, extracellular signal-regulated kinase 1 and 2; GlcN, glucosamine; GM-CSF, granulocyte-macrophage colony-stimulating factor; GROα, growth-related protein α; HIF-2α, hypoxia-inducible factor 2 α; HO-1, heme oxygenase-1; HTRA1, high temperature requirement A serine peptidase 1; HT, hydroxytyrosol; IL, interleukin; iNOS, inducible nitric oxide synthase; JAK2/STAT3, Janus kinase 2/signal transducer and activator of transcription 3; JNK1/2, c-Jun N-terminal kinase 1 and 2; LC-PUFA, long-chain polyunsaturated fatty acid; LPS, lipopolysaccharide; MAPK, mitogen-activated protein kinase; MIP-1α, macrophage inflammatory protein 1α; MMP, matrix metalloproteinase; mTOR, mammalian target of rapamycin; NF-κB, nuclear factor kappa-light-chain-enhancer of activated B cells; Nrf2, nuclear factor erythroid 2-related factor 2; OC, oleocanthal; OP, oleuropein; PAI-1, plasminogen activator inhibitor 1; PERK, protein kinase-like endoplasmic reticulum kinase; PGE2, prostaglandin E2; RUNX, Runt-related transcription factor; ROS, reactive oxygen species; SOCS3, suppressor of cytokine signalling 3; SOD, superoxide dismutase; SIRT, sirtuin; TGF-β, transforming growth factor-β; TIMP-1, tissue inhibitor of matrix metalloproteinase 1; TLR4, toll-like receptor 4; TMJ, temporomandibular joint; TNF-α, tumor necrosis factor α; TUG1, Taurine up-regulated gene 1; VEGF, vascular endothelial growth factor.

Nutraceutical	Ref N.	*In Vitro* Settings (Cell Types and Culture) OA=osteoarthritic N=normal	*In Vivo* Setting	Results	Targeted Pathways
GlcN and CS	[1]	Bovine (steers) N cartilage cultured as explants + IL-1β		GlcN and CS reduce expression of proteolytic enzymes	
GlcN and CS	[2]	Equine N chondrocytes cultured in monolayer + IL-1β		GlcN (but not CS) reduces expression of proteolytic enzymes	**MAPKs (JNK transcription)**
GlcN	[3]	Human N (post-trauma) chondrocytes cultured in monolayer + IL-1β + oncostatin M		GlcN reduces IL-1β expression via epigenetic mechanism (reduced hypomethylation)	**NF-κB**
Hyaluronic acid	[4]	Human OA chondrocytes cultured in monolayer + IL-1α w/wo High Molecular weight (800 and 2700) hyaluronic acid		Hyaluronic acid reduces IL-1α-induced ADAMTS-4 expression and activity	**NF-κB**<br>**MAPKs (ERK1/2)**
ASU	[5]	Bovine N chondrocytes cultured in monolayer		ASU stimulate expression of TGFβ1, TGFβ2 and PAI-1	
ASU	[6]	Human OA chondrocytes cultured in alginate beads with osteoblasts from sclerotic zones		ASU counteract the inhibition of matrix components exerted by OA osteoblasts	
Genistein	[7]	Human N chondrocytes cultured in monolayer, pretreated with genistein prior to LPS exposure		Genistein reduces protein expression of COX and release of NO	
Genistein	[8]	Human OA chondrocytes	Surgically induced OA in rats	Genistein *in vitro* counteracts the IL-1-induced	Nrf2

		in monolayer + IL-1β w/wo genistein	(anterior cruciate ligament transection, ACLT) receiving genistein by diet	expression of iNOS, COX-2, MMPs and stimulates HO-1 expression thus reducing oxidative stress. *In vivo* it delays progression of OA	
Genistein	[9]		Collagenase-induced OA in rats (TMJ OA model) receiving genistein by diet	Genistein *in vivo* counteracts NF-κB activation and reduces expression of IL-1β and TNFα, thus delaying OA progression	**NF-κB**
Omega-3 LC-PUFAs	[10]	Bovine N chondrocytes pre-treated 8 h in the absence or presence of fatty acids, before IL-1α treatment		ALA, EPA and DHA reduce the mRNA level of ADAMTS-4, ADAMTS-5, MMP-3, MMP-13, COX-2, IL-1α, IL-1β and TNF-α	
EPA	[11]	Sodium nitroprusside-induced human N chondrocytes after 8 h pre-incubation with EPA	C57BL/6J mice divided into 4 groups: sham, DMM, DMM + corn oil, and DMM + corn oil + EPA	Intra-articular injection of EPA protects chondrocytes from apoptosis and reduces MMP-13 expression. In *in vitro* study EPA decreases apoptosis and OA markers	**MAPKs (p38)**
DHA	[12]	IL-1β-stimulated human 324 chondrosarcoma SW1353 cells	Rat model of adjuvant-induced arthritis (AIA) +diet containing DHA (50 g/kg)	DHA blocks IL-1β-induced p38 activation	**MAPKs (p38)**
Sulforaphane	[13]	3D cell cultures of GROα-induced human OA chondrocytes. Human C-28/I2 chondrocytes + TNF-α/cycloheximide, or +N(1),N(11)-diethylnorspermine, or +H_2O_2		Sulforaphane protects chondrocytes from oxidative stress and apoptosis induced by inflammatory cytokines, chemokines and other stimuli	**MAPKs (p38)**
Sulforaphane	[14]	Human OA chondrocytes	Surgically induced rat OA model (ACLT) +injection of	Sulforaphane–PLGA microspheres *in vitro* downregulate COX-2, ADAMTS-5 and MMP-2	

			sulforaphane–poly(lactic-co-glycolic acid) (PLGA) microspheres	mRNA. Intra-articular sulforaphane–PLGA microspheres delay the progression of OA	
Sulforaphane	[15]	SW-1353 human chondrosarcoma cell line. Human OA chondrocytes. Fibroblast-like synovial cells from the synovial tissue of OA patients.	DMM on C57BL/6 mice +sulforaphane by diet	Sulforaphane inhibits the expression of key metalloproteinases and blocks inflammation at the level of NF-κB *in vitro* and *in vivo*	**NF-κB**
Sulforaphane	[16]	Human monocyte THP1cell line + 20 ng/ml phorbol myristate acetate to differentiate into matured monocytes/macrophages. Human monocytes from blood of human healthy donors		Sulforaphane has immunomodulatory effect mediated by MAPKs	**MAPKs (ERK1/2, JNK)**
Sulforaphane, allicin, lycopene	[17]	Human OA chondrocytes + H_2O_2		Allicin, sulforaphane and lycopene reduce apoptosis, expression of inflammatory factors, hypertrophic differentiation and enhance chondrogenic matrix synthesis, via Nrf2	Nrf2
OC	[18]	ATDC5 murine chondrogenic cell line + LPS		OC decreases iNOS expression and NO production	
OC	[19]	ATDC5 murine chondrogenic cell line, and J774 macrophages + LPS		OC inhibits LPS-induced NO production in J774 macrophages, decreases MIP-1α and IL-6 mRNA expression and protein synthesis, in both ATDC5 chondrocytes and J774 macrophages. It also inhibits IL-1β, TNF-α and GM-CSF protein synthesis in macrophages	
OC	[20]	Human OA chondrocytes + LPS		OC decreases LPS-mediated inflammatory response	**NF-κB**

			and MMP-13 and ADAMTS-5 induction via MAPKs/NF-kB pathways	**MAPKs (ERK1/2)**
OP	[21]	Human OA chondrocytes + IL-1β	OP suppresses inflammation mediators by MAPKs/NF-kB pathways	**NF-κB** **MAPKs (ERK1/2, p38, JNK)**
HT	[22]	Human OA chondrocytes + H_2O_2, 3D cultures of human OA chondrocytes + GROα	HT decreases apoptosis and gene expression of COX-2, iNOS, MMP-13, RUNX-2 and VEGF	
HT	[23]	C28-I2 chondrocytes and human OA chondrocytes + H_2O_2	HT protects cells by inducing autophagy via SIRT-1 and p62 expression	SIRT-1
HT	[24]	Human OA chondrocytes + H_2O_2	HT decreases miR-9 which targets SIRT-1	SIRT-1
HT	[25]	Rat N chondrocytes + TNF-α	HT protects cells by inducing autophagy via SIRT-6	SIRT-6
HT	[26]	C28-I2 chondrocytes + H_2O_2	HT opposes hypomethylation of miR-9 promoters induced by oxidative stress	SIRT-1
EGCG	[27]	Human OA chondrocytes + IL-1β	EGCG decreases NO and PGE2 production	
EGCG	[28]	Human OA chondrocytes + IL-1β	EGCG inhibits the IL-1β-induced production of NO by interfering with the activation of NF-κB	**NF-κB**
EGCG	[29]	Human OA chondrocytes + AGEs	EGCG inhibits AGE-stimulated cartilage degradation by suppressing MAPKs and NF-κB activation	**NF-κB** **MAPKs (p38, JNK)**
EGCG	[30]	Human OA chondrocytes + IL-1β	EGCG inhibits IL-1β-induced COX-2 expression or PGE2 production via up-regulation of hsa-miR-199a-3p	
Green tea polyphenols	[31]	ATDC5 murine chondrogenic cell line + LPS	Green tea polyphenols decrease apoptosis and proinflammatory cytokine production by p38	**NF-κB** **MAPKs (p38)**

			MAPK and NF-κB
EGCG	[32]	Surgical induction of OA (DMM) on C57BL/6 mice. EGCG (25 mg/kg) or vehicle control was administered daily for four or eight weeks by intraperitoneal injection starting on the day of surgery	EGCG decreases inflammation, cartilage degradation markers and OA-associated pain
Quercetin	[33]	ACLT rat model of OA. At 24 weeks post-operation, intra-articular injection of saline, hydrogel, or hydrogel + quercetin	Quercetin promotes symptom relief and delays the progression of OA
Quercetin and *Ageratum conyzoides L.* extract (containing quercetin)	[34]	70 rats were divided into 14 groups, 7 groups each for DMM- and MIA-induced OA. After 28 days from induction, SHAM and negative group received gel base topically; positive group received sodium diclofenac gel; three-dose group received each 0.84, 1.68, 3.36 mg/g quercetin-loaded nanoparticles gel; and *A. conyzoides L.* group received *A. conyzoides L.* extract gel.	Quercetin-loaded nanoparticle gel and *A. conyzoides L.* extract gel after topic application protect from cartilage degradation
Chondroprote ctive oral formulation	[35]	13 dogs underwent tibial plateau leveling osteotomy and were randomly allocated to treatment (n	Oral supplement provides early improvement of the joint inflammatory microenvironment

containing GlcN, CS, quercetin, vitamin E and Omega-3 LC-PUFAs			6) and control groups (n = 7), the former receiving the oral supplement for 90 days		
Quercetin	[36]		48 rabbits to establish OA model by Hulth modified method (medial parapatellar incision, followed by opening knee joints, transecting anterior cruciate ligaments, and meniscectomy), and subsequently randomized into untreated OA group, celecoxib treated group (celecoxib 100 mg kg-1 by gavage), and quercetin treated group (25 mg kg-1 by gavage).<br>16 non-operated rabbits as the normal controls	Oral administration of quercetin up-regulates SOD and TIMP-1, down-regulates MMP-13, and counteracts cartilage degeneration	
Quercetin	[37]		Meniscal surgery-induced OA rats w/wo quercetin post-treatment (100 mg/Kg/day orally administration)	Quercetin decreases ROS and promotes mitochondrial biogenesis via AMPK/SIRT-1 pathway	SIRT-1<br>AMPK
Quercetin	[38]	Tert-butyl hydroperoxide-stimulated rat chondrocytes	Medial meniscotibial ligament was transected, leading to DMM OA in rats. Immediately after the surgery, quercetin was injected	*In vitro* quercetin inhibits ER stress by activating AMPK/SIRT-1 pathway.<br>*In vivo* it decreases cartilage degeneration and chondrocytes apoptosis	**PERK- eIF2α- CHOP**<br>SIRT-1<br>AMPK

Quercetin	[39]	Rat N chondrocytes +IL-1β +quercetin post-treatment. Co-culture with macrophages	intra-peritoneally<br>18 rats divided into three groups: control group, OA group (by removing the medial meniscus and cutting the anterior meniscotibial ligament), and OA + quercetin group (intra-articular injection)	*In vitro* quercetin reduces inflammation and apoptosis in chondrocytes and modulates synovial macrophages polarization to M2 macrophages promoting cartilage repair. *In vivo* quercetin intra-articular injection decreases OA	**NF-κB** **Akt/mTOR**
Curcumin	[40]	Rabbit articular chondrocytes +AGEs then treated with [illegible]		Curcumin exerts an inhibitory activity on AGE-mediated progression of OA by blocking NF-κB activation	**NF-κB**
Curcumin	[41]	Rat chondrocytes were stimulated with IL-1β to induce an OA-like inflammatory response and treated with curcumin		Curcumin upregulates expression of type II collagen and down-regulates MMP13 expression via IκBα mediated NF-κB inhibition	**NF-κB**
Curcumin	[42]		Intra-articular administration of curcumin in ACTL rats	Curcumin exerts a protective effect by inhibiting TLR4 and its downstream targets	**NF-κB**
Curcumin	[43]		DMM mice subjected to oral administration of curcumin	Curcumin *in vivo* promotes autophagy through modulation of Akt/mTOR pathway, thus contributing to reduced apoptosis and matrix degradation	**Akt/mTOR**
Curcumin	[44]		ACLT rat model of OA subjected to oral administration of curcumin	Curcumin treatment reduces oxidative stress and ER stress by upregulating SIRT-1/PERK- eIF2α-CHOP pathway, thus ameliorating progression of	**PERK- eIF2α- CHOP** SIRT-1
Curcumin	[45]	Human chondrocytes + LPS then treated with curcumin in combination with other compounds		Curcumin exerts a synergistic anti-inflammatory effect in combination with other natural compounds with potential benefits in OA progression	

Curcumin	[46]	Human chondrocytes (HCH-c) and RAW 264.7 cell line in monolayer co-treated with curcumin and lecithin after LPS stimulation		Curcumin and lecithin complex *in vitro* showed anti-inflammatory effects in chondrocytes, thus proving to be potentially beneficial in OA	
Curcumin	[47]	Human TMJ chondrocytes cultured in monolayer and co-treated with IL-1β and curcumin	36 male rats into 3 groups: a degeneration group (injections of complete Freund's adjuvant only), an NS group (weekly injections of NS containing 0.1% DMSO), and a curcumin group (weekly injections of 40 μM curcumin in NS)	Curcumin *in vivo* exerts anti-inflammatory effects in cartilage by reducing major inflammatory mediators of OA via ROS/Nrf2 pathway	Nrf2
Resveratrol	[48]		OA was surgically induced in ACTL rabbit model and resveratrol was injected	Resveratrol intra-articular injection *in vivo* shows protective properties against OA development	
Resveratrol	[49]	Human articular chondrocytes were pre-stimulated with IL-1β and subsequently treated with resveratrol		Resveratrol *in vitro* inhibits in a dose and time-dependent fashion IL-1β –induced activation of apoptosis in OA chondrocytes	
Resveratrol	[50]	Primary human articular chondrocytes, cultured in monolayer and treated with resveratrol		Resveratrol *in vitro* exerts anti-inflammatory and anti-apoptotic effects through the suppression of IL-1β and p53	
Resveratrol	[51]	Healthy human articular chondrocytes were pre-stimulated with resveratrol and co-treated with IL-1β		Resveratrol anti-inflammatory properties occur through the modulation of NF-κB pathway in IL-1β induced *in vitro* OA model	**NF-κB**

Resveratrol	[52]	Human OA chondrocytes in monolayer were pre-stimulated with resveratrol and co-treated with IL-1β		Resveratrol anti-apoptotic effects in OA could be related to inhibition of COX-2 and PGE_2	
Resveratrol	[53]	Pig articular chondrocytes were cultured in monolayer and subsequently treated with resveratrol		Resveratrol *in vitro* protects cartilage from AGEs-dependent degradation by modulation of NF-κB and MAPK pathways	**NF-κB**<br>**MAPKs (p38)**
Resveratrol	[54]	Articular chondrocytes isolated from Wistar rats were transfected with SIRT-1 siRNA and treated with resveratrol		Resveratrol *in vitro* inhibits NF-κB and activates SIRT-1 suggesting a protective role against OA development	**NF-κB**<br>SIRT-1
Resveratrol	[55]	Huma articular OA chondrocytes in monolayer were pre-stimulated with IL-1β before treatment with resveratrol		Resveratrol exerts chondroprotective effects in OA *in vitro* model through modulation of TLR4/NF-κB	**NF-κB**
Resveratrol	[56]		OA was induced surgically (DMM) in C57BL/6 mice subsequently treated with intra-articular injections of resveratrol	Resveratrol *in vivo* protects cartilage from OA via NF-κB/SIRT-1/HIF-2α modulation	**NF-κB**<br>SIRT-1
Resveratrol	[57]		Oral administration of resveratrol in C57BL/6 mice fed with high-fat diet	Resveratrol *in vivo* partially inhibits development of OA induced by high-fat diet	
Resveratrol	[58]	Human articular chondrocytes in monolayer were treated with resveratrol following IL-1β pre-stimulation		Resveratrol *in vitro* exerts chondroprotective effects in OA both in a TLR4-dependent and -independent mechanism	
Resveratrol	[59]		Oral administration of resveratrol in	Resveratrol *in vivo* ameliorates progression of high	

			C57BL/6 mice fed with high-fat diet	fat-induced OA through modulation of TLR4 pathway	
Resveratrol	[60]		Surgical induction (DMM) of OA in C57BL/6 mice subjected to intra-articular injections of resveratrol	Resveratrol *in vitro* concurs to a delay of cartilage degradation in OA through modulation of autophagy	**Akt/mTOR**
Resveratrol	[61]	ATDC5 murine chondrogenic cell line + LPS and subsequently treated with resveratrol		Resveratrol *in vitro* regulates miR-146 thus inhibiting pivotal pathways in LPS-induced inflammatory response in OA	**NF-κB**<br>**MAPKs (p38)**
Resveratrol	[62]	Mice primary articular chondrocytes were isolated and infected with lentivirus targeting SOCS3	Oral administration of resveratrol in C57BL/6 mice fed with high-fat diet	Resveratrol modulation of JAK2/STAT3 pathway ameliorates obesity-related OA	**JAK2/STAT3**
Wogonin	[63]	Rabbit articular chondrocytes +1, 10, 50, or 100 μM of wogonin, followed by incubation in the presence or absence of IL-1β (10 ng/mL) for 24 h	4 groups of rats: control, IL-1β, 50 μM wogonin +IL-1β, 100 μM wogonin +IL-1β.	Wogonin decreases gene expression and production of MMP-3 *in vitro* and *in vivo*.	
Scutellaria baicalensis extract (containing wogonin)	[64]	Human OA chondrocytes pre-treated with fractions of Scutellaria baicalensis extract, then stimulated with IL-1β		Wogonin protects chondrocytes by the suppression of c-Fos/AP-1 activity	
Wogonin	[65]	Human OA chondrocytes pre-treated with wogonin, then stimulated with IL-1β		Wogonin exerts chondroprotective effects through the activation of ROS/ERK/Nrf2 signalling [illegible]	**MAPKs (ERK1/2)**<br>Nrf2
Wogonin	[66]	Human OA chondrocytes		Wogonin localizes in the cell nucleus.	

		pre-treated with wogonin, then stimulated with IL-1β		In *in silico* molecular docking experiments wogonin intercalates between DNA bases	
Wogonin	[67]		Surgical induction of OA (DMM) in C57B6 mice + topical application of cream containing wogonin	Wogonin attenuates OA severity, via MMP-13, NF-κB and HTRA1 decrease	**NF-κB**
Berberine	[68]	Rabbit articular chondrocytes were isolated and treated with various concentration of berberine		Berberine exerts a regulatory function on OA associated genes, such as MMP-3, TIMP-1 and ADAMTS-5	
Berberine	[69]	Healthy rat articular chondrocytes were pre-stimulated with IL-1 β then treated with berberine	Rats were randomly divided into four groups. IL-1 β was used to stimulate OA then berberine was injected in the joints	Berberine exerts anti-catabolic and anti-inflammatory effects on OA development	
Berberine	[70]	Neonatal rat articular cartilage stimulated with IL-1β and treated with increasing concentrations of berberine	ACTL rats were divided into 6 groups including control + low/middle/high dose of intra-articular injections of berberine	Berberine protects articular cartilage from OA-linked damage through Akt signalling pathway	**Akt/mTOR**
Berberine	[71]	Synovial tissue was isolated from OA patients, lately fibroblasts were isolated stimulated with CCN2 and treated with berberine	Collagenase-induced OA (CIOA) rat model, intraperitoneal administration of berberine	Berberine *in vivo* prevents CCN2 inflammatory function, thus preventing cartilage damage in OA model via p38 pathway	
Berberine	[72]	Neonatal rat articular chondrocytes in monolayer were co-treated with increasing doses of berberine and specific inhibitors of the investigated signalling pathways	ACTL rats were divided into 4 groups (low/high dose + control) and subsequently subjected to intra-articular injections of berberine	Berberine reduces NO-induced apoptosis in OA model by modulation of p38/AMPK pathway	**MAPKs (p38)**<br>AMPK

Berberine	[73]		Surgically induced (ACTL + medial menisci resection) OA rats divided in five groups with combinations of intra-articular berberine +chitosan injections	Berberine conjugated with chitosan nanoparticles reduces apoptosis, thus ameliorating OA	
Berberine	[74]	Primary rat articular chondrocytes in monolayer were pre-stimulated with berberine then co-treated with sodium nitroprusside	Surgically induced (ACTL + medial menisci resection) OA rats were divided into 4 grouped (low/high dose + control) and subsequently subjected to intra-articular injections of berberine	Berberine *in vivo* promotes chondrocytes proliferation in SNP-stimulated chondrocytes via Wnt/ β-catenin pathway activation	
Berberine	[75]	Primary rabbit articular chondrocytes co-treated with jasplakinolide to induce reorganization of cytoskeleton and berberine		Berberine *in vitro* cooperates in cytoskeleton reorganization by blocking Akt/p38 mediated differentiation of chondrocytes	**MAPKs (p38)**<br>**Akt/mTOR**
Berberine	[76]	Primary articular chondrocytes isolated from SD rats cultured at 80% confluence were stimulated with IL-1β and treated with berberine		Berberine exerts anti-catabolic and anti-inflammatory properties through inhibition of MAPK- mediated IL-1β inflammatory response	**MAPKs (p38)**
Fisetin	[77]	RAW264.7 cells + LPS		Fisetin exerts anti-inflammatory effects through inhibition of NF-κB and JNK	**NF-κB**<br>**MAPKs (JNK)**
Fisetin	[78]	Human OA chondrocytes pre-treated with fisetin + IL-1β	DMM induction of OA in mice, followed by gavage 20 mg/kg fisetin	*In vitro* fisetin decreases inflammation and degradation markers by maintaining SIRT-1 levels	SIRT-1
Naringin	[79]	Murine N chondrocytes +	DMM induction of OA in mice,	Naringin protects from cartilage degradation by	**NF-κB**

		+ TNF-α	followed by oral administration of naringin	suppressing NF-κB signalling pathway	
Naringin	[80]	RAW 264.7 macrophages + LPS + naringin	Oral administration of naringin before MIA-induced OA procedure in rats	Naringin *in vitro* decreases the production of PGE2, NO, IL-6, and TNF-α. *In vivo* it protects from tissue damage	
Emodin	[81]	ATDC5 murine chondrogenic cell line + LPS + emodin		Emodin decreases apoptosis and inflammatory markers by inhibiting the Notch and NF-κB pathways via up-regulation of lncRNA TUG1	**NF-κB**<br>**Notch**
Emodin	[82]	Rat N chondrocytes pre-treated with emodin + IL-1β	ACLT-induced OA rats + post-treatment of low-concentration and high-concentration of emodin (intra-articular injection)	Emodin protects from cartilage degeneration by inhibiting NF-κB and Wnt/β-catenin signalling	**NF-κB**<br>**Wnt/β-catenin**
Spermidine	[83]	Human N or OA chondrocytes, and HTB-94 chondrosarcoma cells + spermidine.<br>Old/young C57BL/6 mice for *ex vivo* experiments		Spermidine protects from age-related impairment of autophagy	
Spermidine	[84]	Human OA chondrocytes pre-treated with spermidine + H_2O_2		Spermidine protects from DNA damage and NF-κB activation, by autophagy induction	**NF-κB**

Supplementary References:

1. Chan, P.S.; Caron, J.P.; Orth, M.W. Effect of glucosamine and chondroitin sulfate on regulation of gene expression of proteolytic enzymes and their inhibitors in interleukin-1-challenged bovine articular cartilage explants. *Am J. Vet. Res.* **2005**, *66*, 1870–1876.
2. Neil, K.M.; Orth, M.W.; Coussens, P.M.; Chan, P.S.; Caron, J.P. Effects of glucosamine and chondroitin sulfate on mediators of osteoarthritis in cultured equine chondrocytes stimulated by use of recombinant equine interleukin-1beta. *Am. J. Vet. Res.* **2005**, *66*, 1861–1869.
3. Imagawa, K.; de Andres, M.C.; Hashimoto, K.; Pitt, D.; Itoi, E.; Goldring, M.B.; Roach, H.I.; Oreffo, R.O. The epigenetic effect of glucosamine and a nuclear factor-kappa B (NF-kB) inhibitor on primary human chondrocytes--implications for osteoarthritis. *Biochem. Biophys. Res. Commun.* **2011**, *405*, 362–367.

4. Yatabe, T.; Mochizuki, S.; Takizawa, M.; Chijiiwa, M.; Okada, A.; Kimura, T.; Fujita, Y.; Matsumoto, H.; Toyama, Y.; Okada, Y. Hyaluronan inhibits expression of ADAMTS4 (aggrecanase-1) in human osteoarthritic chondrocytes. *Ann. Rheum. Dis.* **2009**, *68*, 1051–1058.
5. Boumediene, K.; Felisaz, N.; Bogdanowicz, P.; Galera, P.; Guillou, G.B.; Pujol, J.P. Avocado/soya unsaponifiables enhance the expression of transforming growth factor beta1 and beta2 in cultured articular chondrocytes. *Arthritis Rheum.* **1999**, *42*, 148–156.
6. Henrotin, Y.E.; Deberg, M.A.; Crielaard, J.M.; Piccardi, N.; Msika, P.; Sanchez, C. Avocado/soybean unsaponifiables prevent the inhibitory effect of osteoarthritic subchondral osteoblasts on aggrecan and type II collagen synthesis by chondrocytes. *J. Rheumatol.* **2006**, *33*, 1668–1678.
7. Hooshmand, S.; Soung do, Y.; Lucas, E.A.; Madihally, S.V.; Levenson, C.W.; Arjmandi, B.H. Genistein reduces the production of proinflammatory molecules in human chondrocytes. *J. Nutr. Biochem.* **2007**, *18*, 609–614.
8. Liu, F.C.; Wang, C.C.; Lu, J.W.; Lee, C.H.; Chen, S.C.; Ho, Y.J.; Peng, Y.J. Chondroprotective Effects of Genistein against Osteoarthritis Induced Joint Inflammation. *Nutrients* **2019**, *11*.
9. Yuan, J.; Ding, W.; Wu, N.; Jiang, S.; Li, W. Protective Effect of Genistein on Condylar Cartilage through Downregulating NF-kappaB Expression in Experimentally Created Osteoarthritis Rats. *Biomed. Res. Int.* **2019**, *2019*, 2629791.
10. Zainal, Z.; Longman, A.J.; Hurst, S.; Duggan, K.; Caterson, B.; Hughes, C.E.; Harwood, J.L. Relative efficacies of omega-3 polyunsaturated fatty acids in reducing expression of key proteins in a model system for studying osteoarthritis. *Osteoarthritis Cartilage* **2009**, *17*, 896–905.
11. Sakata, S.; Hayashi, S.; Fujishiro, T.; Kawakita, K.; Kanzaki, N.; Hashimoto, S.; Iwasa, K.; Chinzei, N.; Kihara, S.; Haneda, M., et al. Oxidative stress-induced apoptosis and matrix loss of chondrocytes is inhibited by eicosapentaenoic acid. *J. Orthop. Res.* **2015**, *33*, 359–365.
12. Wang, Z.; Guo, A.; Ma, L.; Yu, H.; Zhang, L.; Meng, H.; Cui, Y.; Yu, F.; Yang, B. Docosahexenoic acid treatment ameliorates cartilage degeneration via a p38 MAPK-dependent mechanism. *Int J. Mol. Med.* **2016**, *37*, 1542–1550.
13. Facchini, A.; Stanic, I.; Cetrullo, S.; Borzi, R.M.; Filardo, G.; Flamigni, F. Sulforaphane protects human chondrocytes against cell death induced by various stimuli. *J. Cell.* **2011**, *226*, 1771–1779.
14. Ko, J.Y.; Choi, Y.J.; Jeong, G.J.; Im, G.I. Sulforaphane-PLGA microspheres for the intra-articular treatment of osteoarthritis. *Biomaterials* **2013**, *34*, 5359–5368.
15. Davidson, R.K.; Jupp, O.; de Ferrars, R.; Kay, C.D.; Culley, K.L.; Norton, R.; Driscoll, C.; Vincent, T.L.; Donell, S.T.; Bao, Y., et al. Sulforaphane represses matrix-degrading proteases and protects cartilage from destruction in vitro and in vivo. *Arthritis Rheum.* **2013**, *65*, 3130–3140.
16. Pal, S.; Konkimalla, V.B. Sulforaphane regulates phenotypic and functional switching of both induced and spontaneously differentiating human monocytes. *Int. Immunopharmacol.* **2016**, *35*, 85–98.
17. Yang, J.; Song, X.; Feng, Y.; Liu, N.; Fu, Z.; Wu, J.; Li, T.; Chen, H.; Chen, J.; Chen, C., et al. Natural ingredients-derived antioxidants attenuate H2O2-induced oxidative stress and have chondroprotective effects on human osteoarthritic chondrocytes via Keap1/Nrf2 pathway. *Free Radic. Biol. Med.* **2020**, in press.
18. Iacono, A.; Gomez, R.; Sperry, J.; Conde, J.; Bianco, G.; Meli, R.; Gomez-Reino, J.J.; Smith, A.B., 3rd; Gualillo, O. Effect of oleocanthal and its derivatives on inflammatory response induced by lipopolysaccharide in a murine chondrocyte cell line. *Arthritis Rheum.* **2010**, *62*, 1675–1682.
19. Scotece, M.; Gomez, R.; Conde, J.; Lopez, V.; Gomez-Reino, J.J.; Lago, F.; Smith, A.B., 3rd; Gualillo, O. Further evidence for the anti-inflammatory activity of oleocanthal: inhibition of MIP-1alpha and IL-6 in J774 macrophages and in ATDC5 chondrocytes. *Life Sci.* **2012**, *91*, 1229–1235.
20. Scotece, M.; Conde, J.; Abella, V.; Lopez, V.; Francisco, V.; Ruiz, C.; Campos, V.; Lago, F.; Gomez, R.; Pino, J., et al. Oleocanthal Inhibits Catabolic and Inflammatory Mediators LPS-Activated Human Primary Osteoarthritis (OA) Chondrocytes Through MAPKs/NF-kappaB Pathways. *Cell Physiol. Biochem.* **2018**, *49*, 2414–2426.

21. Feng, Z.; Li, X.; Lin, J.; Zheng, W.; Hu, Z.; Xuan, J.; Ni, W.; Pan, X. Oleuropein inhibits the IL-1beta-induced expression of inflammatory mediators by suppressing the of NF-kappaB and MAPKs in human osteoarthritis chondrocytes. *Food Funct.* **2017**, *8*, 3737–3744.
22. Facchini, A.; Cetrullo, S.; D'Adamo, S.; Guidotti, S.; Minguzzi, M.; Borzi, R.M.; Flamigni, F. Hydroxytyrosol prevents increase of osteoarthritis markers in human chondrocytes treated with hydrogen peroxide or growth-related oncogene alpha. *PLoS One* **2014**, *9*, e109724.
23. Cetrullo, S.; D'Adamo, S.; Guidotti, S.; Borzi, R.M.; Flamigni, F. Hydroxytyrosol prevents chondrocyte death under oxidative stress by inducing autophagy through sirtuin 1-dependent and -independent mechanisms. *Biochim. Biophys. Acta* **2016**, *1860*, 1181–1191.
24. D'Adamo, S.; Cetrullo, S.; Guidotti, S.; Borzi, R.M.; Flamigni, F. Hydroxytyrosol modulates the levels of microRNA-9 and its target sirtuin-1 thereby counteracting oxidative stress-induced chondrocyte death. *Osteoarthritis Cartilage* **2017**, *25*, 600–610.
25. Zhi, L.Q.; Yao, S.X.; Liu, H.L.; Li, M.; Duan, N.; Ma, J.B. Hydroxytyrosol inhibits the inflammatory response of osteoarthritis chondrocytes via SIRT6-mediated autophagy. *Mol. Med. Rep.* **2018**, *17*, 4035–4042.
26. D'Adamo, S.; Cetrullo, S.; Borzi, R.M.; Flamigni, F. Effect of oxidative stress and 3-hydroxytyrosol on DNA methylation levels of miR-9 promoters. *J. Cell. Mol. Med.* **2019**, *23*, 7885–7889.
27. Ahmed, S.; Rahman, A.; Hasnain, A.; Lalonde, M.; Goldberg, V.M.; Haqqi, T.M. Green tea polyphenol epigallocatechin-3-gallate inhibits the IL-1 beta-induced activity and expression of cyclooxygenase-2 and nitric oxide synthase-2 in human chondrocytes. *Free Radic. Biol. Med.* **2002**, *33*, 1097–1105.
28. Singh, R.; Ahmed, S.; Islam, N.; Goldberg, V.M.; Haqqi, T.M. Epigallocatechin-3-gallate inhibits interleukin-1beta-induced expression of nitric oxide synthase and production nitric oxide in human chondrocytes: suppression of nuclear factor kappaB activation by degradation of the inhibitor of nuclear factor kappaB. *Arthritis Rheum.* **2002**, *46*, 2079–2086.
29. Rasheed, Z.; Anbazhagan, A.N.; Akhtar, N.; Ramamurthy, S.; Voss, F.R.; Haqqi, T.M. Green tea polyphenol epigallocatechin-3-gallate inhibits advanced glycation end product-induced expression of tumor necrosis factor-alpha and matrix metalloproteinase-13 in human chondrocytes. *Arthritis Res. Ther.* **2009**, *11*, R71.
30. Rasheed, Z.; Rasheed, N.; Al-Shobaili, H.A. Epigallocatechin-3-O-gallate up-regulates microRNA-199a-3p expression by down-regulating the expression of cyclooxygenase-2 stimulated human osteoarthritis chondrocytes. *J. Cell Mol. Med.* **2016**, *20*, 2241–2248.
31. Zhang, Q.; Wang, Y.; Zhang, M.; Ying, H. Green tea polyphenols attenuate LPS-induced inflammation through upregulating microRNA-9 in murine chondrogenic ATDC5 *J. Cell Physiol.* **2019**, *234*, 22604–22612.
32. Leong, D.J.; Choudhury, M.; Hanstein, R.; Hirsh, D.M.; Kim, S.J.; Majeska, R.J.; Schaffler, M.B.; Hardin, J.A.; Spray, D.C.; Goldring, M.B., et al. Green tea polyphenol treatment chondroprotective, anti-inflammatory and palliative in a mouse post-traumatic osteoarthritis model. *Arthritis Res. Ther.* **2014**, *16*, 508
33. Mok, S.W.; Fu, S.C.; Cheuk, Y.C.; Chu, I.M.; Chan, K.M.; Qin, L.; Yung, S.H.; Kevin Ho, K.W. Intra-Articular Delivery of Quercetin Using Thermosensitive Hydrogel Attenuate Cartilage Degradation in an Osteoarthritis Rat Model. *Cartilage* **2018**, 1947603518796550.
34. Permatasari, D.A.; Karliana, D.; Iskandarsyah, I.; Arsianti, A.; Bahtiar, A. Quercetin prevent proteoglycan destruction by inhibits matrix metalloproteinase-9, matrix metalloproteinase-13, a disintegrin and metalloproteinase with thrombospondin motifs-5 expressions on osteoarthritis model rats. *J. Adv. Pharm Technol Res.* **2019**, *10*, 2–8.
35. Martini, F.M.; Brandstetter de Bellesini, A.; Miolo, A.; Del Coco, L.; Fanizzi, F.P.; Crovace, A. Combining a joint health supplement with tibial plateau leveling osteotomy in with cranial cruciate ligament rupture. An exploratory controlled trial. *Int. J. Vet. Sci. Med.* **2017**, *5*, 105-112.
36. Wei, B.; Zhang, Y.; Tang, L.; Ji, Y.; Yan, C.; Zhang, X. Protective effects of quercetin against inflammation and oxidative stress in a rabbit model of knee osteoarthritis. *Drug Res.* **2019**, *80*, 360–367.

37. Qiu, L.; Luo, Y.; Chen, X. Quercetin attenuates mitochondrial dysfunction and biogenesis via upregulated AMPK/SIRT1 signaling pathway in OA rats. *Biomed. Pharmacother.* **2018**, *103*, 1585–1591.
38. Feng, K.; Chen, Z.; Pengcheng, L.; Zhang, S.; Wang, X. Quercetin attenuates oxidative stress-induced apoptosis via SIRT1/AMPK-mediated inhibition of ER stress in rat chondrocytes and prevents the progression of osteoarthritis in a rat model. *J. Cell. Physiol.* **2019**, *234*, 18192–18205.
39. Hu, Y.; Gui, Z.; Zhou, Y.; Xia, L.; Lin, K.; Xu, Y. Quercetin alleviates rat osteoarthritis by inhibiting inflammation and apoptosis of chondrocytes, modulating synovial macrophages polarization to M2 macrophages. *Free Radic. Biol. Med.* **2019**, *145*, 146–160.
40. Yang, Q.; Wu, S.; Mao, X.; Wang, W.; Tai, H. Inhibition effect of curcumin on TNF-alpha and MMP-13 expression induced by advanced glycation end products in *Pharmacology* **2013**, *91*, 77–85.
41. Wang, J.; Ma, J.; Gu, J.H.; Wang, F.Y.; Shang, X.S.; Tao, H.R.; Wang, X. Regulation of type II collagen, matrix metalloproteinase-13 and cell proliferation by interleukin-1beta is mediated by curcumin via inhibition of NF-kappaB signaling in rat chondrocytes. *Mol. Med. Rep.* **2017**, *16*, 1837–1845.
42. Yan, D.; He, B.; Guo, J.; Li, S.; Wang, J. Involvement of TLR4 in the protective effect of intra-articular administration of curcumin on rat experimental osteoarthritis. *Acta Cir. Bras.* **2019**, *34*, e201900604.
43. Zhang, G.; Cao, J.; Yang, E.; Liang, B.; Ding, J.; Liang, J.; Xu, J. Curcumin improves age-related and surgically induced osteoarthritis by promoting autophagy in mice. *Biosci.* **2018**, *38*.
44. Feng, K.; Ge, Y.; Chen, Z.; Li, X.; Liu, Z.; Li, H.; Tang, T.; Yang, F.; Wang, X. Curcumin Inhibits the PERK-eIF2alpha-CHOP Pathway through Promoting SIRT1 Expression in Oxidative Stress-induced Rat Chondrocytes and Ameliorates Osteoarthritis Progression in a Rat Model. *Oxid. Med. Cell Longev.* **2019**, *2019*, 8574386.
45. D'Ascola, A.; Irrera, N.; Ettari, R.; Bitto, A.; Pallio, G.; Mannino, F.; Atteritano, M.; Campo, G.M.; Minutoli, L.; Arcoraci, V., et al. Exploiting Curcumin Synergy With Natural Products Using Quantitative Analysis of Dose-Effect Relationships in an Experimental In Vitro Model of Osteoarthritis. *Front. Pharmacol.* **2019**, *10*, 1347.
46. Kim, L.; Kim, J.Y. Chondroprotective effect of curcumin and lecithin complex in human chondrocytes stimulated by IL-1beta via an anti-inflammatory mechanism. *Food Sci. Biotechnol.* **2019**, *28*, 547–553.
47. Jiang, C.; Luo, P.; Li, X.; Liu, P.; Li, Y.; Xu, J. Nrf2/ARE is a key pathway for curcumin-mediated protection of TMJ chondrocytes from oxidative stress and inflammation. *Cell Stress Chaperones* **2020**, *25*, 395–406.
48. Elmali, N.; Esenkaya, I.; Harma, A.; Ertem, K.; Turkoz, Y.; Mizrak, B. Effect of resveratrol in experimental osteoarthritis in rabbits. *Inflamm. Res.* **2005**, *54*, 158–162.
49. Shakibaei, M.; John, T.; Seifarth, C.; Mobasheri, A. Resveratrol inhibits IL-1 beta-induced stimulation of caspase-3 and cleavage of PARP in human articular chondrocytes in vitro. *Ann. N. Y. Acad. Sci.* **2007**, *1095*, 554–563.
50. Csaki, C.; Keshishzadeh, N.; Fischer, K.; Shakibaei, M. Regulation of inflammation signalling by resveratrol in human chondrocytes in vitro. *Biochem. Pharmacol.* **2008**, *75*, 677–687.
51. Shakibaei, M.; Csaki, C.; Nebrich, S.; Mobasheri, A. Resveratrol suppresses interleukin-1beta-induced inflammatory signaling and apoptosis in human articular chondrocytes: potential for use as a novel nutraceutical for the treatment of osteoarthritis. *Biochem. Pharmacol.* **2008**, *76*, 1426–1439.
52. Dave, M.; Attur, M.; Palmer, G.; Al-Mussawir, H.E.; Kennish, L.; Patel, J.; Abramson, S.B. The antioxidant resveratrol protects against chondrocyte apoptosis via effects on mitochondrial polarization and ATP production. *Arthritis Rheum.* **2008**, *58*, 2786–2797.
53. Liu, F.C.; Hung, L.F.; Wu, W.L.; Chang, D.M.; Huang, C.Y.; Lai, J.H.; Ho, L.J. Chondroprotective effects and mechanisms of resveratrol in advanced glycation end products-stimulated chondrocytes. *Arthritis Res. Ther.* **2010**, *12*, R167.

54. Lei, M.; Wang, J.G.; Xiao, D.M.; Fan, M.; Wang, D.P.; Xiong, J.Y.; Chen, Y.; Ding, Y.; Liu, S.L. Resveratrol inhibits interleukin 1beta-mediated inducible nitric oxide synthase expression in articular chondrocytes by activating SIRT1 and thereby suppressing nuclear factor-kappaB activity. *Eur. J. Pharmacol.* **2012**, *674*, 73–79.
55. Liu, L.; Gu, H.; Liu, H.; Jiao, Y.; Li, K.; Zhao, Y.; An, L.; Yang, J. Protective effect of resveratrol against IL-1beta-induced inflammatory response on human osteoarthritic chondrocytes partly via the TLR4/MyD88/NF-kappaB signaling pathway: an "in vitro study". *Int. J. Mol. Sci* **2014**, *15*, 6925–6940.
56. Li, W.; Cai, L.; Zhang, Y.; Cui, L.; Shen, G. Intra-articular resveratrol injection prevents osteoarthritis progression in a mouse model by activating SIRT1 and thereby silencing HIF-2alpha. *J. Orthop. Res.* **2015**, *33*, 1061–1070.
57. Gu, H.; Li, K.; Li, X.; Yu, X.; Wang, W.; Ding, L.; Liu, L. Oral Resveratrol Prevents Osteoarthritis Progression in C57BL/6J Mice Fed a High-Fat Diet. *Nutrients* **2016**, *8*, 233.
58. Gu, H.; Jiao, Y.; Yu, X.; Li, X.; Wang, W.; Ding, L.; Liu, L. Resveratrol inhibits the IL-1beta-induced expression of MMP-13 and IL-6 in human articular chondrocytes via TLR4/MyD88-dependent and -independent signaling cascades. *Int. J. Mol. Med.* **2017**, *39*, 734–740.
59. Jiang, M.; Li, X.; Yu, X.; Liu, X.; Xu, X.; He, J.; Gu, H.; Liu, L. Oral Administration of Resveratrol Alleviates Osteoarthritis Pathology in C57BL/6J Mice Model Induced by a High-Fat Diet. *Mediators Inflamm.* **2017**, *2017*, 7659023.
60. Qin, N.; Wei, L.; Li, W.; Yang, W.; Cai, L.; Qian, Z.; Wu, S. Local intra-articular injection of resveratrol delays cartilage degeneration in C57BL/6 mice by inducing autophagy AMPK/mTOR pathway. *J. Pharmacol. Sci.* **2017**, *134*, 166–174.
61. Jin, H.; Zhang, H.; Ma, T.; Lan, H.; Feng, S.; Zhu, H.; Ji, Y. Resveratrol Protects Murine Chondrogenic ATDC5 Cells Against LPS-Induced Inflammatory Injury Through Up-Regulating MiR-146b. *Cell. Physiol. Biochem.* **2018**, *47*, 972–980.
62. Jiang, M.; He, J.; Gu, H.; Yang, Y.; Huang, Y.; Xu, X.; Liu, L. Protective effect of resveratrol on obesity-related osteoarthritis via alleviating JAK2/STAT3 signaling pathway is independent of SOCS3. *Toxicol. Appl. Pharmacol.* **2020**, *388*, 114871.
63. Park, J.S.; Lee, H.J.; Lee, D.Y.; Jo, H.S.; Jeong, J.H.; Kim, D.H.; Nam, D.C.; Lee, C.J.; Hwang, S.C. Chondroprotective Effects of Wogonin in Experimental Models of in vitro and in vivo. *Biomol. Ther. (Seoul)* **2015**, *23*, 442–448.
64. Khan, N.M.; Haseeb, A.; Ansari, M.Y.; Haqqi, T.M. A wogonin-rich-fraction of Scutellaria baicalensis root extract exerts chondroprotective effects by suppressing IL-1beta-induced activation of AP-1 in human OA chondrocytes. *Sci. Rep.* **2017**, *7*, 43789.
65. Khan, N.M.; Haseeb, A.; Ansari, M.Y.; Devarapalli, P.; Haynie, S.; Haqqi, T.M. Wogonin, a plant derived small molecule, exerts potent anti-inflammatory and effects through the activation of ROS/ERK/Nrf2 signaling pathways in human Osteoarthritis chondrocytes. *Free Radic. Biol. Med.* **2017**, *106*, 288–301.
66. Khan, N.M.; Ahmad, I.; Ansari, M.Y.; Haqqi, T.M. Wogonin, a natural flavonoid, intercalates with genomic DNA and exhibits protective effects in IL-1beta stimulated osteoarthritis chondrocytes. *Chem. Biol. Interact.* **2017**, *274*, 13–23.
67. Smith, J.F.; Starr, E.G.; Goodman, M.A.; Hanson, R.B.; Palmer, T.A.; Woolstenhulme, J.B.; Weyand, J.A.; Marchant, A.D.; Bueckers, S.L.; Nelson, T.K., et al. Topical Application Wogonin Provides a Novel Treatment of Knee Osteoarthritis. *Front. Physiol.* **2020**, *11*, 80.
68. Moon, P.D.; Jeong, H.S.; Chun, C.S.; Kim, H.M. Baekjeolyusin-tang and its active component berberine block the release of collagen and proteoglycan from IL-1beta-stimulated rabbit cartilage and down-regulate matrix metalloproteinases in rabbit chondrocytes. *Phytother. Res.* **2011**, *25*, 844–850.
69. Hu, P.F.; Chen, W.P.; Tang, J.L.; Bao, J.P.; Wu, L.D. Protective effects of berberine in an experimental rat osteoarthritis model. *Phytother. Res.* **2011**, *25*, 878–885.
70. Zhao, H.; Zhang, T.; Xia, C.; Shi, L.; Wang, S.; Zheng, X.; Hu, T.; Zhang, B. Berberine ameliorates cartilage degeneration in interleukin-1beta-stimulated rat chondrocytes and in rat model of osteoarthritis via Akt signalling. *J. Cell. Mol. Med.* **2014**, *18*, 283–292.
71. Liu, S.C.; Lee, H.P.; Hung, C.Y.; Tsai, C.H.; Li, T.M.; Tang, C.H. Berberine attenuates CCN2-induced IL-1beta expression and prevents cartilage degradation in a rat model of osteoarthritis. *Toxicol. Appl. Pharmacol.* **2015**, *289*, 20–29.

72. Zhou, Y.; Liu, S.Q.; Yu, L.; He, B.; Wu, S.H.; Zhao, Q.; Xia, S.Q.; Mei, H.J. Berberine prevents nitric oxide-induced rat chondrocyte apoptosis and cartilage degeneration in a rat osteoarthritis model via AMPK and p38 MAPK signaling. *Apoptosis* **2015**, *20*, 1187–1199.
73. Zhou, Y.; Liu, S.Q.; Peng, H.; Yu, L.; He, B.; Zhao, Q. In vivo anti-apoptosis activity of novel berberine-loaded chitosan nanoparticles effectively ameliorates osteoarthritis. *Int. Immunopharmacol.* **2015**, *28*, 34–43.
74. Zhou, Y.; Tao, H.; Li, Y.; Deng, M.; He, B.; Xia, S.; Zhang, C.; Liu, S. Berberine promotes proliferation of sodium nitroprusside-stimulated rat chondrocytes and osteoarthritic cartilage via Wnt/beta-catenin pathway. *Eur. J. Pharmacol.* **2016**, *789*, 109–118.
75. Yu, S.M.; Cho, H.; Kim, G.H.; Chung, K.W.; Seo, S.Y.; Kim, S.J. Berberine induces dedifferentiation by actin cytoskeleton reorganization via phosphoinositide 3-kinase/Akt and p38 kinase pathways in rabbit articular chondrocytes. *Exp. Biol. Med. (Maywood)* **2016**, *241*, 800–807.
76. Li, X.; He, P.; Hou, Y.; Chen, S.; Xiao, Z.; Zhan, J.; Luo, D.; Gu, M.; Lin, D. Berberine inhibits the interleukin-1 beta-induced inflammatory response via MAPK downregulation rat articular chondrocytes. *Dru. Dev. Res.* **2019**, *80*, 637–645.
77. Kim, S.C.; Kang, S.H.; Jeong, S.J.; Kim, S.H.; Ko, H.S. Inhibition of c-Jun N-terminal kinase and nuclear factor kappa B pathways mediates fisetin-exerted anti-inflammatory activity in lipopolysccharide-treated RAW264.7 cells. *Immunopharmacol. Immunotoxicol.* **2012**, *34*, 645–650.
78. Zheng, W.; Feng, Z.; You, S.; Zhang, H.; Tao, Z.; Wang, Q.; Chen, H.; Wu, Y. Fisetin inhibits IL-1beta-induced inflammatory response in human osteoarthritis chondrocytes through activating SIRT1 and attenuates the progression of osteoarthritis in mice. *Int. Immunopharmacol.* **2017**, *45*, 135–147.
79. Zhao, Y.; Li, Z.; Wang, W.; Zhang, H.; Chen, J.; Su, P.; Liu, L.; Li, W. Naringin Protects Against Cartilage Destruction in Osteoarthritis Through Repression of NF-kappaB Signaling Pathway. *Inflammation* **2016**, *39*, 385–392.
80. Xu, Q.; Zhang, Z.F.; Sun, W.X. Effect of Naringin on Monosodium Iodoacetate-Induced Osteoarthritis Pain in Rats. *Med. Sci Monit* **2017**, *23*, 3746-3751.
81. Liang, Z.; Ren, C. Emodin attenuates apoptosis and inflammation induced by LPS through up-regulating lncRNA TUG1 in murine chondrogenic ATDC5 cells. *Biomed. Pharmacother.* **2018**, *103*, 897–902.
82. Ding, Q.H.; Ye, C.Y.; Chen, E.M.; Zhang, W.; Wang, X.H. Emodin ameliorates cartilage degradation in osteoarthritis by inhibiting NF-kappaB and Wnt/beta-catenin signaling in-vitro and in-vivo. *Int Immunopharmacol* **2018**, *61*, 222–230.
83. Sacitharan, P.K.; Lwin, S.; Gharios, G.B.; Edwards, J.R. Spermidine restores dysregulated autophagy and polyamine synthesis in aged and osteoarthritic chondrocytes via EP300. *Exp. Mol. Med.* **2018**, *50*, 123.
84. D'Adamo, S.; Cetrullo, S.; Guidotti, S.; Silvestri, Y.; Minguzzi, M.; Santi, S.; Cattini, L.; Filardo, G.; Flamigni, F.; Borzi, R.M. Spermidine rescues the deregulated autophagic response to oxidative stress of osteoarthritic chondrocytes. *Free Radic. Biol. Med.* **2020**, *153*, 159–172.
